# The LRRK2 Variant E193K Prevents Mitochondrial Fission Upon MPP+ Treatment by Altering LRRK2 Binding to DRP1

**DOI:** 10.3389/fnmol.2018.00064

**Published:** 2018-02-28

**Authors:** Maria Perez Carrion, Francesca Pischedda, Alice Biosa, Isabella Russo, Letizia Straniero, Laura Civiero, Marianna Guida, Christian J. Gloeckner, Nicola Ticozzi, Cinzia Tiloca, Claudio Mariani, Gianni Pezzoli, Stefano Duga, Irene Pichler, Lifeng Pan, John E. Landers, Elisa Greggio, Michael W. Hess, Stefano Goldwurm, Giovanni Piccoli

**Affiliations:** ^1^Dulbecco Telethon Institute, CIBIO, Università degli Studi di Trento, Trento, Italy; ^2^Dipartimento di Biologia, Università di Padova, Padova, Italy; ^3^Humanitas Clinical and Research Center, Milan, Italy; ^4^Institute for Biomedicine, Eurac Research, Affiliated Institute of the University of Lübeck, Bolzano, Italy; ^5^German Center for Neurodegenerative Diseases (DZNE), Tübingen, Germany; ^6^Institute for Ophthalmic Research, Center for Ophthalmology, University of Tübingen, Tübingen, Germany; ^7^Dino Ferrari Center, Department of Pathophysiology and Transplantation, University of Milan, Milan, Italy; ^8^Department of Neurology and Laboratory of Neuroscience, Istituto Auxologico Italiano, Milan, Italy; ^9^Parkinson Institute, ASST Gaetano Pini-CTO, Milan, Italy; ^10^Department of Biomedical Sciences, Humanitas University, Milan, Italy; ^11^Shanghai Institute of Organic Chemistry, Chinese Academy of Sciences, Shanghai, China; ^12^Department of Neurology, University of Massachusetts Medical School, University of Massachusetts, Worcester, MA, United States; ^13^Division of Histology and Embryology, Innsbruck Medical University, Innsbruck, Austria; ^14^Department of Neuroscience Rita Levi Montalcini, University of Turin, Turin, Italy

**Keywords:** LRRK2, DRP1, mitochondria, protein interaction, Parkinson’s disease

## Abstract

Mutations in leucine-rich repeat kinase 2 gene (*LRRK2*) are associated with familial and sporadic Parkinson’s disease (PD). LRRK2 is a complex protein that consists of multiple domains, including 13 putative armadillo-type repeats at the N-terminus. In this study, we analyzed the functional and molecular consequences of a novel variant, E193K, identified in an Italian family. E193K substitution does not influence LRRK2 kinase activity. Instead it affects LRRK2 biochemical properties, such as phosphorylation at Ser935 and affinity for 14-3-3ε. Primary fibroblasts obtained from an E193K carrier demonstrated increased cellular toxicity and abnormal mitochondrial fission upon 1-methyl-4-phenylpyridinium treatment. We found that E193K alters LRRK2 binding to DRP1, a crucial mediator of mitochondrial fission. Our data support a role for LRRK2 as a scaffolding protein influencing mitochondrial fission.

## Introduction

Parkinson’s disease (PD) is an age-related disorder that affects 2% of the population above 65-years. PD is related to the progressive loss of dopaminergic neurons in the *substantia nigra* (Moore et al., 2005; Poewe et al., 2017) and is clinically characterized by bradykinesia, rigidity and resting tremor. Although the majority of cases do not correlate with clear genetic causes, mutations in the Leucine-rich repeat kinase 2 (LRRK2) gene (PARK8; OMIM 609007) have been unequivocally related to late-onset PD. LRRK2 mutations have been identified in up to 13% of familial PD cases (Paisán-Ruíz et al., 2004; Zimprich et al., 2004) and also account for 1%–2% of not familial cases (Aasly et al., 2005; Goldwurm et al., 2005). LRRK2 protein includes some functional domains such as, from N- to C-terminus armadillo, ankyrin, the namesake leucine-rich repeats, a ROC GTPase domain (Ras of complex proteins), a COR dimerization domain (C-terminal of ROC), a kinase domain and WD40 repeats (Bosgraaf and Van Haastert, 2003; Mills et al., 2012). Genetic and functional analyses have correlated several single nucleotide variants falling in different LRRK2 domains to PD (Paisán-Ruiz et al., 2013) but only five missense mutations within the ROC, COR and kinase domains segregate with PD, being the kinase hyper-activating G2019S mutation the most common. Emerging data suggest the relevance of domains outside the LRRK2 enzymatic core. In fact, the characterization of the G2385R substitution in the WD40 domain as a pathological variant (Tan, 2006; Tan et al., 2009) that affects LRRK2 biochemical properties (Rudenko et al., 2012) and binding to synaptic vesicles (Piccoli et al., 2014; Carrion et al., 2017) indicates the relevance of LRRK2 domains devoid of enzymatic activities. Here we investigated the functional impact of a novel missense variant identified in an Italian family with three siblings affected by PD, E193K. E193K falls within the N-terminus, where a cluster of LRRK2-specific repeats organized as variants of the armadillo repeat structure have been identified (Marín, 2006; Mills et al., 2012). We found that the E193K variant affects LRRK2 supra-molecular organization, binding to DRP1 and cellular and mitochondrial response to 1-methyl-4-phenylpyridinium (MPP+).

## Materials and Methods

### Subjects

We studied one non-consanguineous family originating from Southern-Italy with three siblings affected by PD out of 10, and no history of neurological diseases in the previous generations. Additional DNA samples were obtained from the Parkinson Institute Biobank: 429 familial PD (at least on first or second degree relative affected), 179 early-onset PD (onset <40 years of age), 167 PD cases from the same geographical area of the index family (Calabria); 960 healthy controls (age at withdrawal 65 years ± 7). The clinical diagnosis of PD was established according to the UK Brain Bank criteria (Hughes et al., 1992, 2001). Patients derived fibroblasts were obtained from the Parkinson Institute Biobank (part of the Telethon Genetic Biobank Network http://biobanknetwork.telethon.it/). This study was approved by the Ethical Committee “Comitato Etico Milano Area C” (http://comitatoeticoareac.ospedaleniguarda.it/) on the 26/06/2015 (Numero Registro dei pareri: 370-062015) and was conducted according to the Declaration of Helsinki and to the Italian legislation on sensitive personal data recording. Written informed consent was obtained from all subjects.

### Exome Sequencing

Genomic DNA was isolated from peripheral blood with standard protocols. Exome sequencing was performed in two affected individuals (G-0502 and G-1350) using an exome array (SeqCap EZ Human Exome Library v2.0, Nimblegen) adapted for sequencing on the Illumina HiSeq2000 platform. Alignment of short reads sequences to the human genome (hg19) was obtained with BWA (Li and Durbin, 2009) and variant detection was performed with the GATK software package (McKenna et al., 2010) according to best practice recommendations. Quality control and filtering of candidate variants were performed using an in-house pipeline (Wu et al., 2012). All novel variants identified through exome sequencing and segregating with PD in the family were subsequently screened first on a panel of aged-matched Italian healthy controls (*n* = 960) and then in an Italian cohort of healthy subjects (*n* = 1769) recruited within the “Atherosclerosis, Thrombosis, and Vascular Biology Italian Study Group” (ATVB), as previously described (Atherosclerosis, Thrombosis, and Vascular Biology Italian Study Group, 2003).

### Mutation Analysis

The screening for the E193K variant was performed amplifying a 212bp region surrounding the mutation (primers available on request), and the obtained PCR products were analyzed by high-resolution melting (HRM) analysis using a LightCycler 480 (Roche, Basel, Switzerland). Samples that presented an abnormal melting curve, compatible with a heteroduplex formation, were subsequently sequenced on an ABI 3130XL sequencer (Thermo Scientific, Waltham, MA, USA).

### Cell Cultures

Human fibroblasts were collected from one PD patient with E193K LRRK2 mutation who allowed skin biopsy (G-1350) and two age- and sex-matched healthy controls and G2019S carriers. Primary fibroblasts were banked at Cell Line and DNA Biobank from patients affected by Genetic Diseases and provided at passages 1–2. Cells were grown in Dulbecco’s Modified Eagle Medium (DMEM; Invitrogen, Carlsbad, CA, USA) supplemented with 20% FBS (Invitrogen, Carlsbad, CA, USA), 1% L-glutamine and 1% penicillin/streptomycin (Gibco, Thermo Fisher). Primary cells were used for all experiments with less than 10 passages *in vitro*. N2A (ATCC CCL-131), HEK293 (ATCC CRL-1573) and HeLa cells (ATCC CL-2) were grown in DMEM with 10% FBS, 1% L-glutamine and 1% penicillin/streptomycin. Cells were maintained at a density of 1 × 10^6^ in a 75 cm^2^ tissue culture flask (Corning, New York, NY, USA) and incubated at 37°C under saturating humidity in 5% CO_2_.

### Plasmids and Transfection

Human LRRK2 armadillo domain (aa 1-397) was subcloned into pDEST15 (N-terminal GST tag) using the Gateway system (Life Technologies) as described previously (Piccoli et al., 2014). Full-length myc-hLRRK2, GFP-hLRRK2, RFP-hLRRK2 or Strep-FLAG-hLRRK2 hLRRK2 and Strep-FLAG 14-3-3ε constructs were already described (Gloeckner et al., 2006, 2007). The E193K variant was generated in both vectors by site-directed mutagenesis using the QuikChange mutagenesis kit (Stratagene). Strep-FLAG LRRK2 K1906M and Strep-FLAG LRRK2 913-2527 (hereinafter termed as ΔN-terminal) were amplified by PCR from the LRRK2 cDNA and cloned into pDEST Strep-FLAG (N-SF-TAP) plasmid as already described (Gloeckner et al., 2006). Strep-FLAG LRRK2 1-2141 (from now on termed as ΔWD40) was already described (Piccoli et al., 2014). GFP-DRP1 plasmid was kindly provided by Dr. Van der Bliek (Smirnova et al., 2001). N2a, HEK293, and HeLa cells were transfected by lipofection using Lipofectamine 2000 (Life Technologies) following manufacturer’s instructions and processed 2 days after. Human fibroblast transfection was performed with Amaxa™ Basic Nucleofector™ Kit (Lonza). Briefly, for each transfection, 1 × 10^6^ cells were resuspended in 100 μL of nucleofector solution for mammalian fibroblast and added 3 μg of GFP or GFP-tagged DRP1 plasmid. The mix was transferred to the cuvette and nucleofection was performed using A33 programme on the Amaxa apparatus. Then 500 μL of warm medium were added to the cells that were plated on 12-mm coverslips for 24 h before further treatment.

### Pull-down, Immuno-precipitation, Filter Retardation Assay, Western-Blotting and Antibodies

LRRK2 GST-fusion domains and GST were expressed in the *E. coli* BL21 strain (Life Technologies) and purified as described earlier. Briefly, 5 μg of each GST fusion protein was loaded onto glutathione-sepharose resin (GE-Healthcare, Freiburg) and co-incubated with adult mouse brain lysate (1 mg of total protein). Beads were washed twice with high salt buffer (300 mM NaCl, 50 mM Tris-HCl, pH 7.4) and samples were eluted in Laemmli Buffer 2× for 10 min at 55°C.

For LRRK2 pull-down from heterologous cell lines, 2 days after transfection cells were solubilized in lysis buffer (150 mM NaCl, 2 mM EDTA, 50 mM Tris-HCl, 1% NP-40 and 0.25% sodium deoxycholate, pH 7.4) completed with protease and phosphatase inhibitors (Calbiochem) for 1 h at 4°C. LRRK2 was precipitated using Strep-Tactin Superflow resin (Iba) for 2 h at 4°C. Washing conditions were performed with high salt buffer (300 mM NaCl, 50 mM Tris-HCl pH 7.4) and interacting proteins were eluted in Laemmli buffer 2× at 55°C for 10 min. Pull-down efficiency was judged as the amount of preys normalized to bait quantity as monitored by Ponceau staining. In immuno-precipitation assays, 4 μg of rat anti-LRRK2 (LANK/24D8 clone) antibody or rat anti-IgG (Abcam) was incubated with 1 mg of protein lysate and loaded onto protein G-sepharose resin (GE-Healthcare, Freiburg). In both procedures, resins were extensively washed in Tris-EDTA buffer (10 mM Tris-HCl, pH8.0, 1 mM EDTA, 150 mM NaCl, 0.2% Triton-X100) and samples eluted with Laemmli buffer 2×. In the filter retardation assay, proteins were extracted on PBS 1× plus protease and phosphatase inhibitors (Calbiochem) and bath-sonicated for 6 s. Protein amount was quantified by Bradford’s method and samples were prepared diluting 10 μg of total protein in 120 μl of PBS 1× and filtered through a cellulose acetate membrane and through a nitrocellulose membrane (both from GE Healthcare) by using a 96-well vacuum dot blot system. For protein identification by Western blotting, samples were loaded onto 4%–12% NuPAGE gels (Invitrogen); the proteins were transferred onto nitrocellulose membrane (GE Healthcare) at 82V for 120 min at 4°C. In all procedures, the primary antibodies were applied overnight in blocking buffer (20 mM Tris, pH 7.4, 150 mM NaCl, 0.1% Tween 20, and 5% non-fat dry milk). Primary antibodies (source in parenthesis) included: rabbit anti-LRRK2 1:500 (MJFF2, c41-2), rabbit anti-pSer935 LRRK2 (UDD2-10-12, University of Dundee), mouse anti-DRP1 1:1000 (clone 8, BD Transduction Systems Abcam), rabbit anti-pSer616 DRP1 1:1000 (Cell Signalling), anti-mitofusin 2 (N-terminal) 1:1000 (Sigma), mouse anti-α-Tubulin 1:3000 (Sigma), mouse anti-β-Actin 1:4000 (Sigma), mouse anti-Flag 1:1000 (Sigma). Membranes were washed three times for 10 min with TBS-Tween buffer. The secondary antibodies HRP-conjugated anti-rabbit or anti-mouse (Jackson Immunoresearch) were used at 1:7000 dilution. Proteins were detected using the ECL prime detection system (GE Healthcare). Images were acquired with the ChemiDoc Touch imaging system (BioRad), and protein quantification was performed measuring the optical density of the specific bands with ImageJ software (NIH).

### MTT Reduction Assay

The 3-(4,5-dimethylthiazol-2-yl)-2,5-diphenyltetrazolium bromide (MTT) assay developed by Mosmann (Mosmann, 1983) was performed to measure the cytotoxic effect of MPP^+^. Human fibroblasts were seeded in a 96-well plate at a concentration of 5 × 10^3^ cell/cm^2^ and incubated at 37°C for 24 h. Then, cells were treated with MPP^+^ at different concentrations (1 mM or 3 mM; Sigma) or fresh medium for 24 h (control condition). To perform the assay, MTT was solubilized at a concentration of 5 mg/ml in PBS 1× and added to a final concentration of 0.25 mg/ml in medium. Cells were incubated for 2 h at 37°C. Then, the medium was decanted, and formazan precipitates were solubilized in 200 μL of DMSO. The absorbance was measured at 570 nm using a spectrophotometer. Cell viability was expressed setting the control condition as 100% of vitality.

### Mitochondrial Morphology, ROS Production Measurement and LRRK2 Cellular Distribution

To analyze mitochondrial morphology, human fibroblasts and Hela cells were seeded on 12-mm glass coverslips and incubated at 37°C for 24 h. After transfection, when indicated, cells were treated with MPP+ (1 mM) or fresh medium for 24 h and then incubated with 500 nM MitoTracker Red CMXRos (Molecular Probes, Invitrogen) for 30 min at 37°C. Mitochondrial superoxide content in fibroblasts was characterized by incubation with 2.5 μM Mitosox Red (Molecular Probes, Invitrogen) for 45 min at 37°C. To study LRRK2 cellular localization, RFP-tagged LRRK2 wild-type (WT) or E193K and GFP vectors were overexpressed in N2A cells for 48 h. In all cases, after incubation period cells were washed with warm PBS 1× and fixed with 4% paraformaldehyde for 10 min at room temperature. For nuclear staining, DAPI was prepared at 1:3000 proportion in PBS 1× and incubated for 15 min at room temperature.

Coverslips were mounted with prolonged reagent (Life Technologies). Images were acquired with Zeiss Observer Z1 microscope equipped with Apotome module using an EC Plan-Neofluar 40×/0.75 Oil objective, pixel size 0.114 μm × 0.114 μm or a Plan-Apochromat 100×/1.40 objective, pixel size 0.045 μm × 0.045 μm. The obtained images provide an axial resolution comparable to confocal microscopy (Schaefer et al., 2004; Garini et al., 2005). Cells were randomly chosen in at least four independent experiments for each condition. All the measurements were performed with ImageJ software (NIH) using mitochondrial morphology plugin (developed by Ruben K. Dagda at the University of Pittsburgh). Parameters were automatically determined and expressed as mitochondrial content (percentage of cytosol occupied by mitochondria) and index of interconnectivity (area/perimeter ratio of mitochondria). Reactive oxygen species (ROS) content was quantified with ImageJ software analyzing the integrated density of the fluorescent signal.

### Electron Microscopy

Fibroblasts were plated at days 10–12 after thawing onto sapphire coverslips and cultured for 2–3 days until ~70%–80% confluence. The samples were cryo-fixed using high-pressure freezing, followed by freeze-substitution and epoxy resin embedding for thin-section transmission electron microscopy as previously described (Studer et al., 1989; Hess et al., 2000).

### Characterization of Mitochondrial Activity with High-Resolution Respirometry

The activity of the mitochondrial respiration in fibroblasts was measured using a high-resolution respirometer (Oxygraph-2k; Oroboros Instruments, Innsbruck, Austria) as previously described (Pesta and Gnaiger, 2012). Briefly, oxygen consumption was measured in cells permeabilized with digitonin (8 μg/ml) in MiRo5 medium (10 mM KH_2_PO_4_, 60 mM lactobionic acid, 20 mM HEPES, 3 mM MgCl_2_, 0.5 mM EGTA, 20 mM taurine, 110 mM D-Sucrose and 1 mg/ml BSA fatty acid free). For the characterization of mitochondrial respiration the following characteristics were assessed: (1) physiological respiratory activity in intact cells (routine respiration); (2) complex I-, (3) complex II–dependent respiration, and (4) maximal capacity of the respiratory chain (MAX: not physiological maximal uncoupled respiration that is not limited by the enzyme activity of the ATP synthase) after induction by stepwise titration of carbonyl cyanide p-(trifluoromethoxy) phenylhydrazone, FCCP (0.5 μM). Residual oxygen consumption, ROX (respiration attributable to other cellular oxygen-consuming processes besides the respiratory chain) was measured after addition of 2.5 μM antimycin A. ROX was subtracted from the other parameters. Absolute respiration values were normalized for the total number of cells (800 × 10^3^/chamber). All reagents were purchased from Sigma Aldrich.

### Kinase and PhosTag Assays

*In vitro* kinase assay was perfomed as described (Civiero et al., 2015). Briefly, HEK-293T cells transfected with Strep-Flag LRRK2 WT, E193K or K1906M and with 3xFlag-Rab8, were lysed in buffer A [20 mM Tris-HCl pH 7.5, 150 mM NaCl, 1 mM EDTA, Na_4_O_7_P_2_ 2.5 mM, -glycerophosphate 1 mM, NaVO_4_ 1 mM, 0.5% Tween 20, Protease inhibitor cocktail, 1× Complete Mini Protease Inhibitor Cocktail (Roche Applied Science)]. Cleared lysates (1 ml) were incubated with anti-FLAG-M2-agarose beads (Sigma) by rotating overnight at 4°C. Resin complexes were washed with different buffers (twice with 20 mM Tris–HCl, 500 mM NaCl, 0.5% Tween 20; twice with 20 mM Tris–HCl, 300 mM NaCl, 0.5% Tween 20; twice with 20 mM Tris–HCl, 150 mM NaCl, 0.5% Tween 20; twice with 20 mM Tris–HCl, 150 mM NaCl, 0.1% Tween; and twice with 20 mM Tris–HCl, 150 mM NaCl, 0.02% Tween 20) and proteins were eluted in kinase buffer (25 mM Tris-HCl pH 7.5, 5 mM β-glycerophosphate, 2 mM dithiothreitol, 0.1 mM Na_3_VO_4_, 10 mM MgCl_2_, Tween 0.02% and 150 ng/μl of 3X-FLAG peptide) for 45 min at 4°C with shaking. Eluted proteins were resolved by SDS–PAGE and stained with Coomassie G-250 to verify protein purity, Rab8 and LRRK2 protein concentration was calculated by densitometry, using a standard curve with bovine serum albumin (BSA). Phosphorylation reactions were performed in kinase buffer in presence of LRRK2 20 nM and Rab8 300 nM (where indicated) and 1 mM ATP at 30°C for 1 h in a final volume of 30 μl. Reactions were terminated with 4× Laemmli buffer and by boiling at 95°C for 10 min. Samples were separated by SDS-PAGE or taking advantage of PhosTag gel to discriminate phosphorylation status as previously described (Civiero et al., 2017). Briefly, to perform PhosTag analysis, samples were supplemented with 10 mM MnCl_2_ before loading gels. Gels for Phos-tag Tris-glycine polyacrylamide gels consisted of a stacking gel [4% (w/v) acrylamide, 125 mM Tris/HCl, pH 6.8, 0.1% (w/v) SDS, 0.2% (v/v) TEMED and 0.08% (w/v) APS] and a separating gel [7.5% (w/v) acrylamide, 375 mM Tris/HCl, pH 8.8, 0.1% (w/v) SDS, 75 μM Phos-tag acrylamide, 150 μM MnCl_2_, 0.1% (v/v) TEMED and 0.05% (w/v) APS]. After eletrophoretic separation, samples were transferred onto PVDF membranes (Bio-Rad) using the Trans-Blot Turbo Transfer System (Bio-Rad) in semi-dry conditions using Trans-Blot Turbo Transfer Buffer (Bio-Rad) in 20% (v/v) ethanol at 25 V for 20 min. Membranes were blocked for 40 min in 5% (w/v) skimmed milk in TBS-T buffer (20 mM Tris-HCl pH 7.4, 150 mM NaCl, 0.1% (v/v) Tween-20), followed by incubation for 1 h with an antibody against the LRRK2 autophosphorylation sites Thr2483 (MJF-R8, Abcam, 1:2000) and Ser1292 (Abcam, 1:2000), total LRRK2 (MJFF2, Abcam 1:2000) and FLAG epitope (Sigma, 1:1000). Membranes were washed four times for 10 min with TBS-T buffer and incubated for 1 h at room temperature with HorseRadish-Peroxidase (HRP)-conjugated rabbit secondary antibodies (1:15,000) in 5% (w/v) skimmed milk in TBS-T buffer. Membranes were washed in TBS-T buffer followed by visualization using ECL Western Blotting Detection Reagents (GE Healthcare). Densitometry analysis carried out using ImageJ software was performed and data were expressed as phospho/total LRRK2 protein levels.

### Size Exclusion Chromatography

Lysates (0.5 ml) from WT and E193K fibroblasts were separated on a Superose 6 10/300 column (Ge Healthcare, Waukesha, WI, USA) pre-equilibrated with 20 mM Tris-HCl pH 7.5, 150 mM NaCl and 0.06% (v/v) Triton X-100. The flow rate used was 0.5 ml/min. Fractions of 0.25 ml were collected, and 1 μl spotted onto a nitrocellulose membrane and analyzed by dot blot. The membrane was blocked with 10% milk in TBS plus 0.1% Tween-20 (TTBS) and incubated with rabbit monoclonal anti-LRRK2 (MJFF2, Abcam, 1:2000) and subsequently, HRP-conjugated rabbit secondary antibodies (1:15,000) in TTBS with 10% milk. Immunoproteins were visualized using ECL (GE, Healthcare, Waukesha, WI, USA).

### Homology Modeling

Modeling templates were identified in the Protein Data Bank (Berman et al., 2002) using the profile-profile alignment program Phyre2 (Kelley et al., 2015). The initial homology model was build based on the ARM domain structure of adenomatous polyposis coli (APC) protein from human (PDB code: 3T7U). The model structure was further refined using the YASARA energy minimization server to increase the model accuracy (Krieger et al., 2002). The quality of final structure model was validated using PROCHECK (Laskowski et al., 1996). PyMOL^1^ was used for visualization of the final model.

### Statistical Analysis

All data are expressed as the mean ± standard error of the mean (SE). All data were logged into PRISM and were analyzed with an unpaired Student’s *T*-test (two classes) or ANOVA followed by Tuckey’s *post hoc* test (more than two classes). The indications of the number of experiment (n) and the level of significance (p) are indicated throughout the text.

## Results

### Identification of LRRK2 E193K Variant

**Figure 1:**
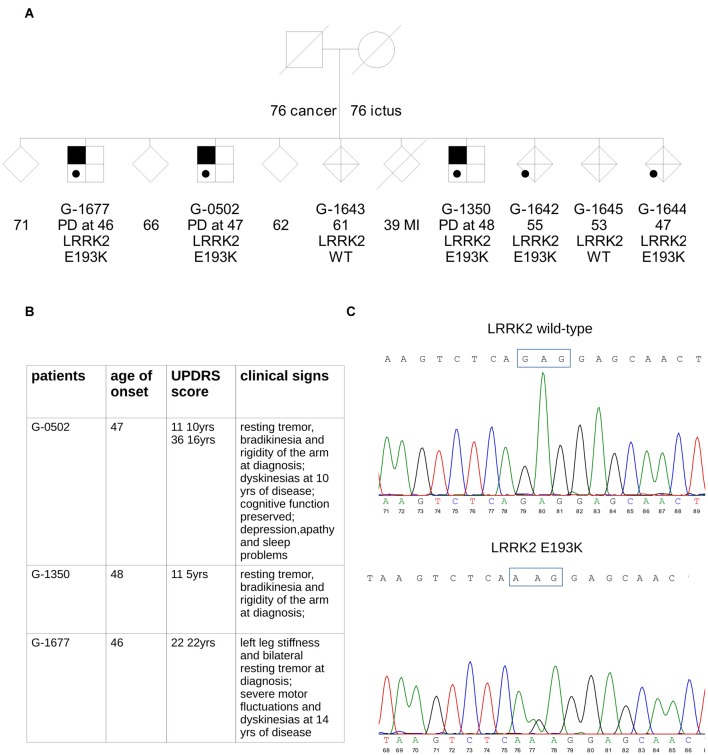
Simplified pedigree of the analyzed family. **(A)** Symbols with black upper corner indicate individuals affected by Parkinson’s disease (PD). Age of onset of PD (years) is reported; in the patients’ relatives, age at last examination or age at death is reported. The result of leucine-rich repeat kinase 2 (LRRK2) genetic screening for the mutation was either wild type (WT) or heterozygous carrier (E193K). Gender of healthy siblings has been masked to protect the anonymity of the families. **(B)** The table summarizes clinical data of the three E193K carriers. **(C)** Sequencing of PCR product from exon 6 of WT and mutant alleles. The upper chromatogram of the portion of the sequencing gel shows the wild allele and the lower the heterozygous mutant allele.

We performed exome sequencing in an Italian family with three affected siblings (Figure 1A).

In the three brothers, symptoms were typical of idiopathic PD with onset around 46–48 years in all cases (Figure 1B). Progression was slow, and none developed cognitive decline. A more detailed clinical description is reported in supplementary material. Two of the three PD cases in the family (G-0502, G-1350) are being followed at the Parkinson Center of Milan. Analysis of the major PD genes did not reveal any pathogenic mutation: Parkin, PINK1, DJ-1, SNCA, GBA, LRRK2 (exon 31 and 41). These same two patients were analyzed by whole-exome sequencing. One interesting novel variant in the LRRK2 gene, c.577G>A, causing the missense change p.E193K, was present, in the heterozygous state, in all three affected siblings (Figure 1C). We tested this variant in the other relatives: the same G>A transition was present as well in two healthy relatives (G-1642 and G-1644) suggesting incomplete penetrance as reported for the other LRRK2 mutations identified so far (Goldwurm et al., 2011). This variant is absent from public mutation databases (GnomAD Browser) and ethnically matched controls (about 1800 Italian exomes).

### The E193K Variant Affects LRRK2 Biochemical Features

**Figure 2:**
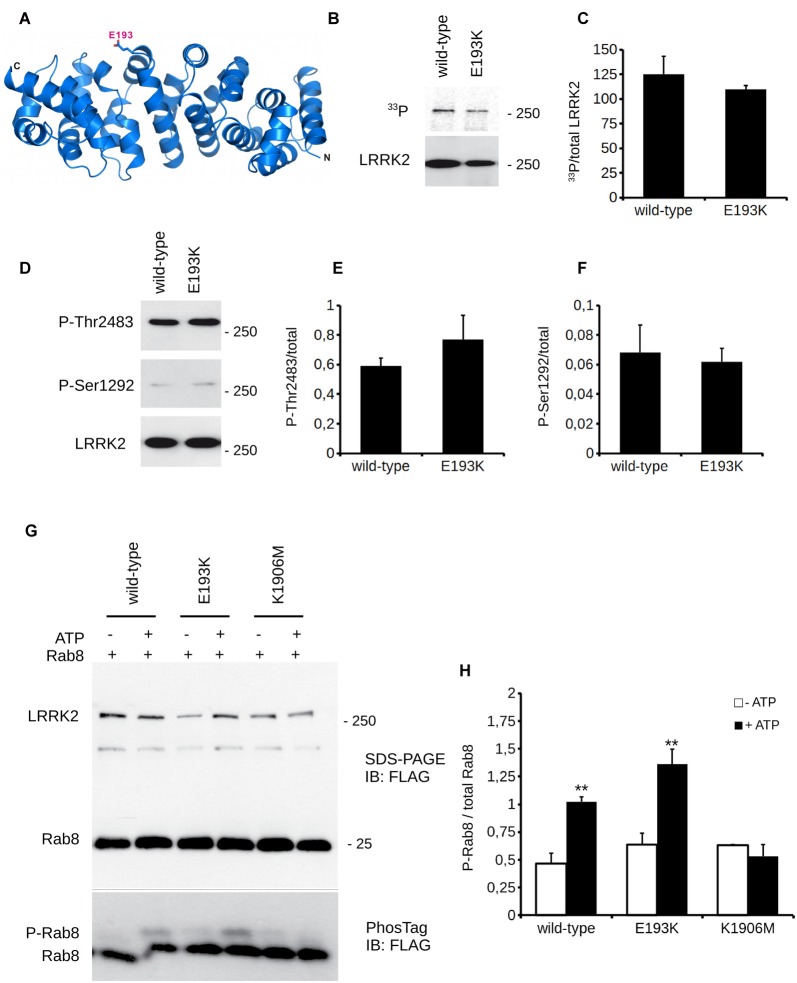
E193K variant does not affect LRRK2 kinase activity. **(A)** Ribbon diagram showing a structural homology model (based on the ARM domain of adenomatous polyposis coli (APC) protein from human, PDB: 3T7U) for the LRRK2 N-terminal region (residues 7-322). N-terminus (N) and C-terminus (C) of the structural model as well as the position of E193 (stick model) are indicated. **(B)** *In vitro* radioactive kinase assays of 3x-Flag LRRK2 WT and E193K purified from HEK cells. Five nanomolar of purified Flag-LRRK2 WT or E193K from HEK293T cells were subjected to *in vitro* radiometric kinase assays and the radioactivity incorporated was quantified by PhosphoImager. Upper panel represents autophosphorylation and lower panel western blotting with anti-flag antibodies to quantify total loading. **(C)** Quantification of moles of ^33^P incorporated by LRRK2. Graphs report mean ± standard error (SE), *n* = 4. **(D)** *In vitro* kinase assays as in **(A)**. Five nanomolar of purified Flag-LRRK2 WT or E193K from HEK293T cells were also subjected to *in vitro* non-radioactive kinase assays. Autosphosphorylation levels were measured by western blotting with anti-Ser1292 and anti-Thr2483 antibodies (upper panels). Lower panel represents total protein loading, probed with anti-flag antibodies. **(E)** Quantification of phosphorylation at Thr2483 and **(F)** Ser1292, expressed as optical density and normalized vs. total LRRK2 protein amount. Graphs report mean ± SE, *n* = 4. **(G)** Purified Flag-LRRK2 WT, E193K or K1906M protein were incubated with Flag-Rab8 (1:15 molar ratio) in the presence or absence of 1 mM ATP and subjected to PhosTag assay to analyze phosphorylation stoichiometry and SDS-PAGE to verify total protein amount. Anti-Flag antibody was used to reveal LRRK2 and Rab8. **(H)** Quantification of Rab8 phosphorylation, expressed as optical density and normalized vs. total Rab8 (phosphorylated + unphosphorylated band). Data are presented as mean ± SE (*n* = 3); ** *p* < 0.01 vs. -ATP, same LRRK2 variant.

**Figure 3:**
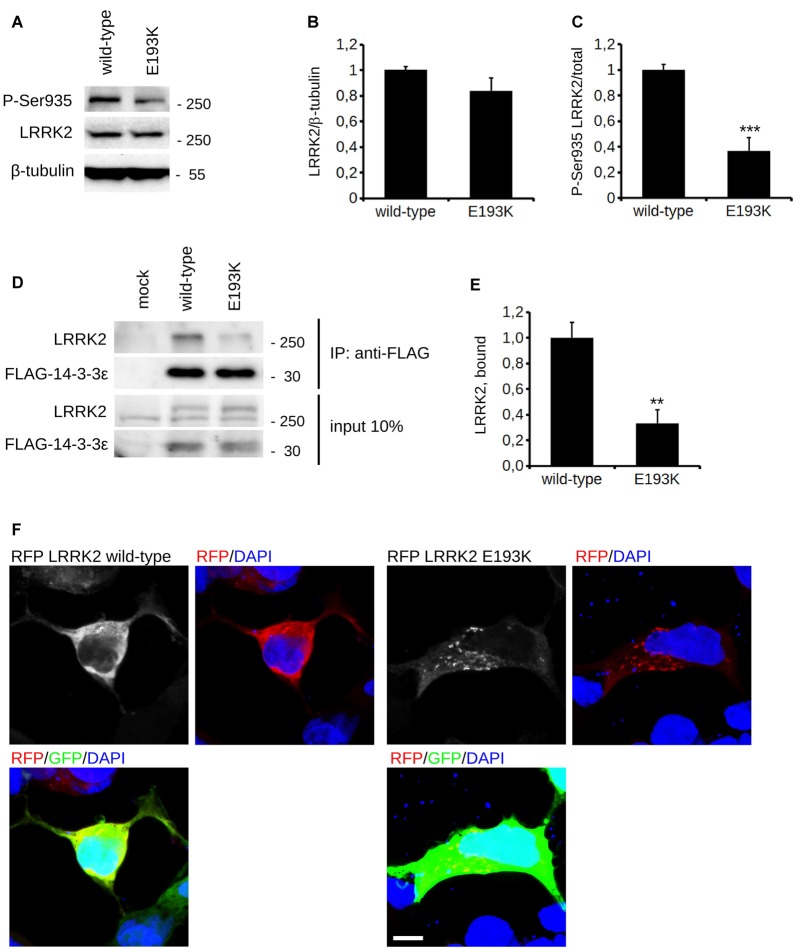
E193K variant affects LRRK2 phosphorylation at Ser935. **(A)** Protein lysate prepared from primary fibroblast obtained from healthy individuals or E193K carrier was analyzed by western-blotting to appreciate total LRRK2 level and phosphorylation at Ser-935. **(B,C)** The graphs report LRRK2 total level, expressed as optical density and normalized vs. β-tubulin amount **(B)** and P-Ser935 level, expressed as optical density and normalized vs. total LRRK2 amount **(C)**. Data are shown as mean ± SE, *n* = 8; ***p < 0.001 vs. WT. **(D)** N2a cells expressing FLAG-14-3-3ε together with myc-LRRK2 WT or myc-LRRK2 E193K variant were solubilized and processed for FLAG immunopurification. We evaluated the extent of LRRK2 binding to 14-3-3ε by measuring the amount of myc LRRK2 co-precipitating with FLAG 14-3-3ε **(E)** The graph reports the amount of myc-LRRK2 variant recovered in FLAG immunoprecipitates. Data were normalized to the amount of 14-3-3ε immunoprecipitated and expressed as mean ± SE, *n* = 4; **p < 0.01versus WT. **(F)** N2a cells expressing GFP together with RFP-LRRK2 WT or RFP-LRRK2 E193K variant were processed for imaging purposes. Scale bar = 10 μm.

**Figure 4:**
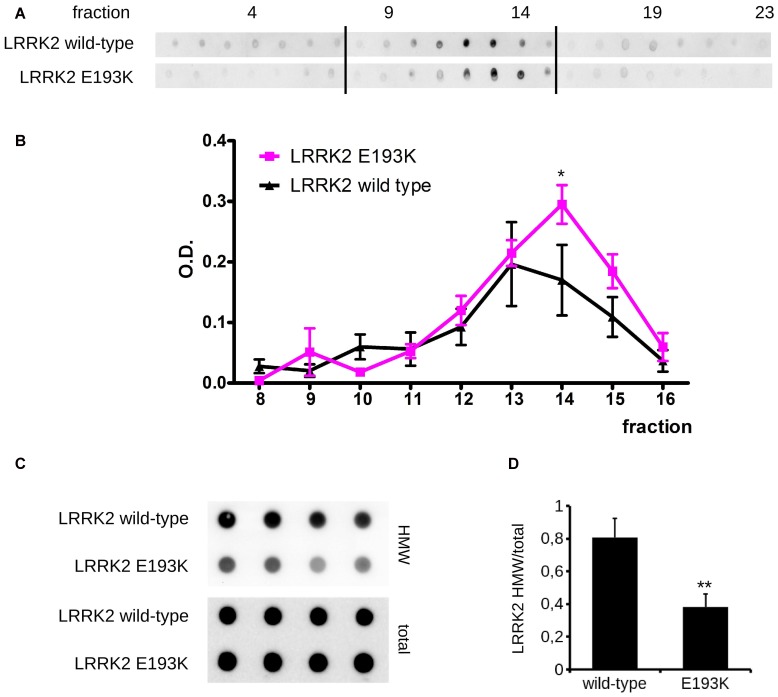
E193K variant affects LRRK2 supra-molecular organization. **(A)** Protein lysate prepared from primary fibroblast obtained from healthy individuals or E193K carrier was separated by size exclusion chromatography (SEC). LRRK2 elution profile was revealed by dot-blot using anti-LRRK2 antibody. Theoretical molecular weight are: V_0_ at fraction 8.5, 669 kDa at fraction 12, 449 kDa at fraction 13. **(B)** The graph reports the intensity of each dot (fraction) normalized by the integrated intensities. Data are shown as mean ± SE, *n* = 4; *p < 0.05 vs. WT. Column void volume is 7.5 ml. **(C)** Primary fibroblasts obtained from healthy individuals or an E193K carrier were assayed by filter retardation assay to isolate high molecular weight (HMW) form of LRRK2 on acetate cellulose membrane and total LRRK2 on nitrocellulose membrane. **(D)** The graph reports LRRK2 HMW amount expressed as fold over total LRRK2. Data are shown as mean ± SE, *n* = 8; **p < 0.01 vs. WT.

*In silico* prediction suggests that the highly conserved E193 residue is located within the armadillo repeats domain. Structural modeling for the armadillo repeats indicates that E193 residue is solvent-exposed (Figure 2A). Being LRRK2 a kinase, we first investigated the potential impact of E193K variant on LRRK2 catalytic properties. Autophosphorylation is a recognized output of LRRK2 specific kinase activity (Greggio et al., 2008; Sheng et al., 2012). Thus, we compared the autophosphorylation activity of LRRK2 WT vs. E193K variant. We immunoprecipitated FLAG-LRRK2 variants from transfected HEK293 cells and performed *in vitro* radiometric phosphorylation assays. LRRK2 WT and E193K variant possess comparable autophosphorylation levels as judged by total P33 incorporation (Figures 2B,C). We obtained similar results investigating by western-blotting two specific LRRK2 autophosphorylation sites, Ser1292 and Thr2483 (Sheng et al., 2012; Figures 2D–F). Rab8 is a well established substrate of LRRK2 (Steger et al., 2016). Thus we evaluated the efficiency of the phosphorylation of LRRK2 WT, E193K and the artificial kinase-dead variant K1906M on Rab8 with PhosTag gels coupled with immunoblot analysis. We found that LRRK2 WT and E193K variant phosphorylates Rab8 protein in a similar extent *in vitro* after 60 min incubation under kinase assay conditions, as revealed by the retarded electrophoretic mobility of phosphorylated Rab8 (Figures 2G,H). These data suggest that E193K has no major impact on LRRK2 kinase activity. Phosphorylation at Ser935 is altered in the presence of multiple PD mutations (Nichols et al., 2010). Therefore, we profiled LRRK2 phosphorylation at Ser935 in WT and E193K primary fibroblasts. Western-blotting analysis showed that while LRRK2 total levels do not differ substantially between the two lines, phospho-Ser935 level is impaired in fibroblast carrying the E193K variant (Figures 3A–C). We obtained a similar result by western-blotting analysis of LRRK2 Ser935 phosphorylation in N2A cells upon over-expression of LRRK2 variants (Supplementary Figures S1A,B). Phosphoresidues at Ser910/935 stabilize LRRK2 binding to 14-3-3s chaperons (Dzamko et al., 2010). Given the impact of E193K substitution on Ser935 phosphorylation, we investigated the effect of E193K on LRRK2 binding to 14-3-3ε. To this aim, we expressed myc-tagged LRRK2 WT or E193K variant together with FLAG-14-3-3ε in N2A cells (Gloeckner et al., 2007). After solubilization, we immobilized 14-3-3ε on anti-FLAG beads, and we studied the amount of bound LRRK2 variant by western-blotting. Noteworthy, E193K demonstrated a significantly reduced affinity for 14-3-3ε compared to WT LRRK2 (Figures 3D,E). 14-3-3 binding to phosphorylated Ser935 influences LRRK2 sub-cellular distribution (Nichols et al., 2010). Thus, we studied the cellular localization of RFP LRRK2 WT and E193K variant upon over-expression in N2A cells. We noticed that while LRRK2 WT was diffuse within the cytosol, E193K variant appeared in perinuclear clusters (Figure 3F). The armadillo repeats domain allows interaction with other proteins: E193K variant may, therefore, affect LRRK2 homo and hetero interaction. To test this hypothesis, we analyzed LRRK2 E193K elution profile by means of size exclusion chromatography (SEC) experiments in samples prepared from primary fibroblasts derived from an E193K carrier or healthy subjects (Figure 4A). Interestingly, the elution profile of LRRK2 E193K substantially differs from the one of WT LRRK2, with the appearance of a robust peak in the low-molecular weight fractions (fraction 14, Figure 4B). To complement this evidence, we performed a filter retardation assay to detect the presence of high molecular weight (HMW) protein complexes including LRRK2. By western-blotting, we detected a substantial reduction of HMW LRRK2-positive complex in specimen prepared from E193K fibroblasts (Figures 4C,D). Altogether these data suggest that the E193K substitution impacts LRRK2 supra-molecular organization.

### E193K Variant Affects Mitochondrial Response to MPP+

**Figure 5:**
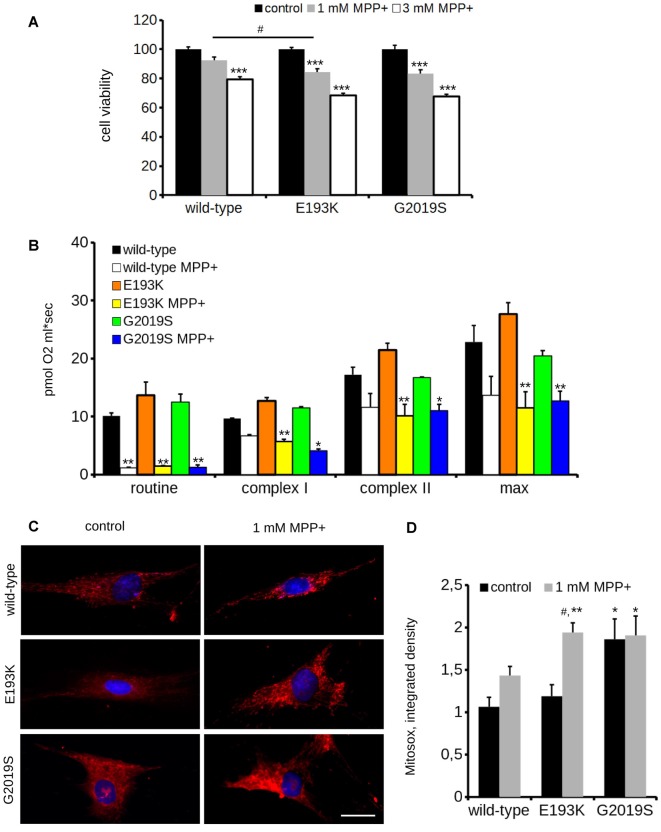
E193K mitochondria are more sensitive to MPP+. **(A)** Primary fibroblasts obtained from healthy individuals, E193K or G2019S carriers were treated with 1 or 3 mM MPP+ for 24 h and cell vitality was measured by the means of MTT assays. The graph reports relative viability. Data are shown as mean ± SE, *n* = 6; ***p < 0.001 vs. control conditions, same genotype, ^#^*p* < 0.05 vs. WT, same treatment. **(B)** Oxygen consumption assessed by high resolution respirometry of primary fibroblasts after 24 h treatment with 1 mM MPP+. The graph reports routine activity (routine respiration), complex I and complex II—dependent activity (upon treatment with malate, glutamate and succinate) and maximal activity (maximal uncoupled respiration after FCCP injection). Data show means ± SE, *n* = 4; *, **p < 0.05, 0.01 vs. control conditions, same genotype. **(C)** Primary fibroblasts obtained from healthy individuals, G2019S or E193K carriers were treated with 1 mM MPP+ for 24 h and then incubated with Mitosox to measure the reactive oxygen species (ROS) production. Scale bar = 25 μM. **(D)** The graph reports the intensity of the Mitosox signal, expressed as integrated density. Data are expressed as mean ± SE, *n* = 4 (20 cells/experiment); ^#^*p* < 0.05 vs. control condition, same genotype. *, **p < 0.05, 0.01 vs. WT, same treatment.

**Figure 6:**
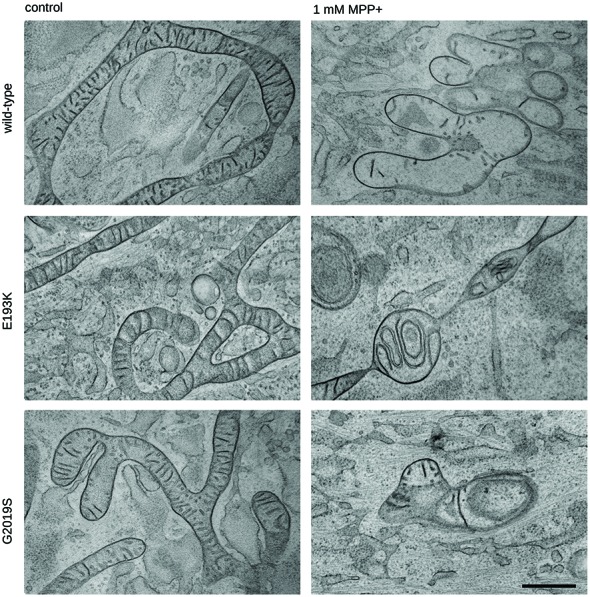
Mitochondrial morphology is impaired in E193K fibroblasts. Transmission electron microscopy from cryo-fixed, freeze-substituted fibroblast cultures. Untreated WT, G2019S or E193K fibroblasts showed elongated, partly branched mitochondria with correctly arranged cristae. Upon treatment with MPP+, WT and G2019S fibroblasts appeared consistently fragmented (and locally highly swollen), showing reduced and disorganized cristae. By contrast, E193K fibroblasts were regularly characterized by conspicuously segmented, highly anastomosing mitochondrial networks and altered cristae morphology. Representative images are shown from three independent cell culture experiments (20 cells genotype/treatment/experiment). Scale bar = 500 nm.

One of the most recognized pathological events in PD is mitochondrial dysfunction, and LRRK2 has been proposed to be associated with mitochondrial biology (Biskup et al., 2006; Verma et al., 2017). LRRK2 influences mitochondrial dynamics (Wang et al., 2012) and response to toxins (Saha et al., 2009). Therefore, we analyzed the impact of the LRRK2 E193K variant on cell viability upon exposure to the mitochondrial toxin MPP+. To this aim, we treated primary fibroblasts obtained from healthy individuals, E193K and G2019S carriers with increasing concentrations of MPP+ and we evaluated cell viability by MTT assays (Figure 5A). The treatment with 3 mM MPP+ triggered cell death in all cell lines despite the different genotype. Noteworthy, we noticed that 1 mM MPP+ did not result in overt toxicity in WT fibroblasts but resulted noxious for E193K and, as expected, for G2019S cells (Yakhine-Diop et al., 2014). MPP+ accumulates in mitochondria (Ramsay and Singer, 1986), and high concentrations of MPP+ partially inhibit mitochondrial complex I activity (Nicklas et al., 1985). Therefore, we examined the mitochondrial respiration in primary fibroblasts using a high-resolution respirometer (Figure 5B and Supplementary Figure S1F). Fibroblasts from healthy subjects, E193K or G2019S carriers showed an almost similar respiratory activity under basal conditions. Upon 1 mM MPP+ treatment, we measured a robust drop of routine respiration in all lines. We noticed subtle differences among the different genotypes while analyzing complex I and II and maximal respiration activity. In particular, E193K and G2019S lines were more sensitive to MPP+ in terms of complex I and II and maximal respiratory capability. Aberrant functioning of the mitochondrial respiratory chain can induce the production of noxious ROS. Therefore, we measured ROS production in basal conditions and upon 1 mM MPP+ insult. To this aim, we incubated primary fibroblasts with MitoSOX Red, a fluorogenic dye selective for mitochondrial superoxide (Figure 5C). Interestingly, while 1 mM MPP+ treatment did not result in an overt increase of ROS production in control or G2019S fibroblasts, it almost doubled ROS level in E193K cells (Figure 5D). MPP+ affects mitochondrial structural organization (Zhu et al., 2007). For the reliable preservation of the dynamically changing mitochondrial ultrastructure, we chose rapid cryo-fixation through use of high-pressure freezing, instead of conventional chemical fixation. In WT and G2019S fibroblasts, MPP+ induced dramatic alterations in mitochondrial morphology. Mitochondria appeared locally highly dilated, consistently showing disorganized cristae. Furthermore, the mitochondrial shape had changed from branched tubules (as observed in untreated samples) to spherical and rod-shaped fragments. In untreated E193K fibroblasts, size and shape of mitochondria were almost normal. However, upon MPP+ insult, mitochondria in E193K fibroblasts appeared as segmented, highly anastomosing networks with partially disorganized cristae (Figure 6 and Supplementary Figure S2), indicating impaired fission. Altogether, these data suggest that the E193K variant influences functional and structural mitochondrial response to toxins.

### E193K Variant Alters LRRK2 Affinity for DRP1

**Figure 7:**
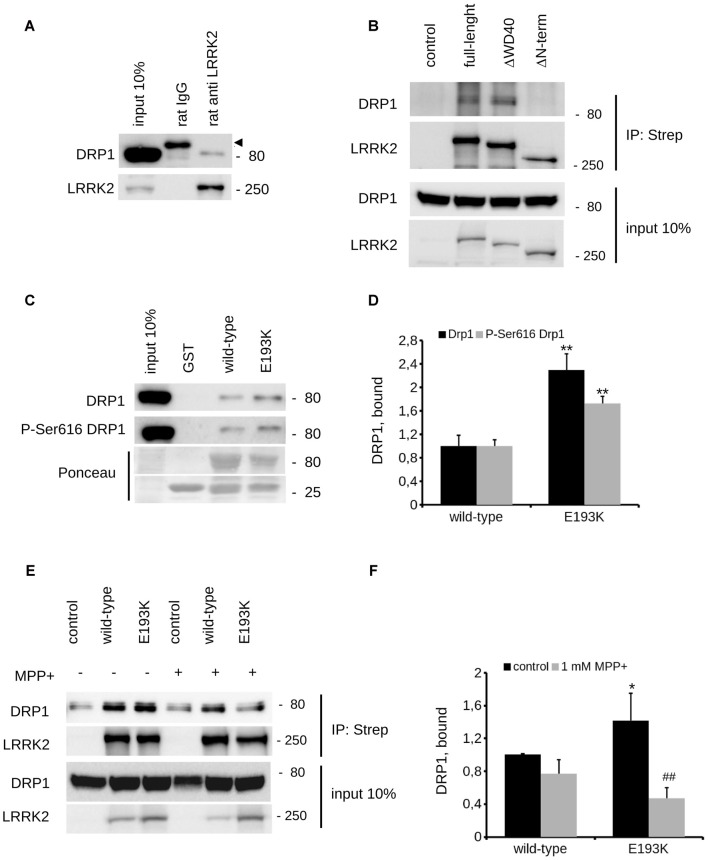
E193K variant affects LRRK2-DRP1 complex. **(A)** Extracts of mouse adult forebrain were incubated with anti-LRRK2 antibodies or rat IgG. The immunocomplexes were isolated with protein G-Sepharose and the samples were resolved by SDS-PAGE and analyzed by immunoblotting with anti DRP1 and anti LRRK2 antibodies. Arrowhead indicates unspecific band recognized by anti-rabbit secondary antibody. **(B)** We isolated on streptavidin resin strep-FLAG-LRRK2 full-length (full-length), strep-FLAG-LRRK2ΔWD40 (ΔWD40) and strep-FLAG-LRRK2ΔN–terminal (ΔN–terminal) protein from HEK293 over-expressing cells. Interacting proteins were resolved by western-blotting. **(C)** We performed a GST-pull down approach to explore the interactome associated to LRRK2 N-terminal Armadillo domain. GST-fusion proteins corresponding to Armadillo domain of LRRK2 WT and LRRK2 E193K (E193K) were used to retain interactors from adult forebrain lysate. The complexes were isolated with GSH-Sepharose beads, the samples were resolved by SDS-PAGE and analyzed by immunoblotting with anti DRP1 and anti P-Ser616 DRP1 antibodies. **(D)** We evaluated the extent of DRP1 and P-Ser616 DRP1 bound to WT and E193K Armadillo domain expressed as ratio over WT domain. Graph reports mean ± SE; *n* = 4; **p < 0.01 vs. WT. **(E)** N2A cells expressing Strep-FLAG-LRRK2 WT or Strep-FLAG-LRRK2 E193K variant were treated or not with MPP+ (1 mM, 24 h) solubilized and processed for streptavidin immunopurification. We evaluated the extent of DRP1 binding to LRRK2 by measuring the amount of DRP1 protein co-precipitating with Strep-FLAG LRRK2 variant. **(F)** The graph reports the amount of DRP1 recovered in FLAG immunoprecipitates. Data were normalized to the amount of LRRK2 variant immunoprecipitated and expressed as mean ± SE, *n* = 7; *p < 0.05 vs. WT, ^##^*p* < 0.01 vs. E193K not treated.

**Figure 8:**
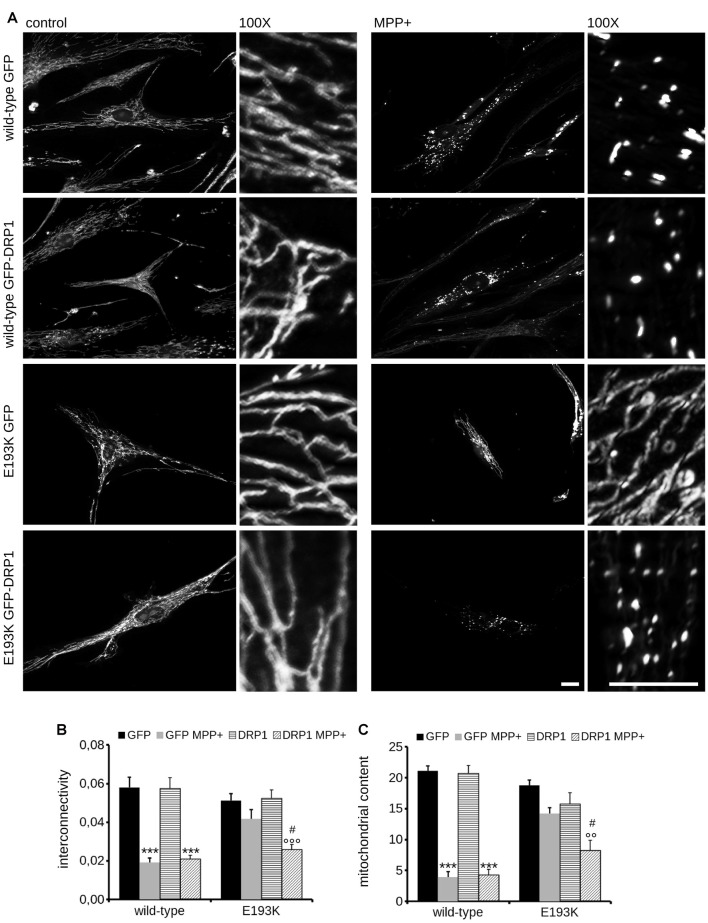
Mitochondrial fission upon MPP+ is impaired in E193K fibroblasts. **(A)** Primary fibroblasts obtained from healthy individuals and an E193K carrier were transfected with GFP or GFP-DRP1, treated with 1 mM MPP+ for 24 h and then incubated with MitotrackerRed to investigate mitochondrial morphology. Scale bars = 10 μm. **(B,C)** The graphs report mitochondrial interconnectivity **(B)** and content **(C)**. Data are expressed as mean ± SE (*n* = 4, 5–6 cells/experiment); °°*p* < 0.01 vs. control treatment, same transfection, ***,°°°*p* < 0.001 vs. control treatment, same transfection, ^#^*p* < 0.05 vs. GFP MPP+.

Mitochondria react to toxic insult by activating a cycle of fusion-fission events (Wang et al., 2011). Among the panel of proteins regulating mitochondrial fission, DRP1 plays a major role (Chang and Blackstone, 2010). DRP1 has been suggested as a LRRK2 interactor (Wang et al., 2012). First, we confirmed LRRK2-DRP1 interaction by immunoprecipitation of endogenous LRRK2 from mouse brain lysate (Figure 7A). LRRK2 harbors at least two major sites for protein interaction featured by its N- and C-terminus (Carrion et al., 2017). Thus, we mapped the LRRK2 domain hosting the interaction with DRP1. To this aim, we expressed Strep-FLAG LRRK2 full-length (full length), Strep-FLAG LRRK2 missing the N-terminal fragment (ΔN-terminal) and Strep-FLAG LRRK2 missing the C-terminal WD40 domain (ΔWD40) in HEK293 cells. Upon streptavidin-pull-down, we studied interacting proteins by western-blotting. Interestingly, we noticed that the interaction with DRP1 was severely reduced in the LRRK2 ΔN-terminal variant (Figure 7B). Next, we investigated whether the E193K variant might affect LRRK2-DRP1 interaction taking advantage of a domain-wise pull-down approach. To this aim, we expressed LRRK2 Arm WT and E193K domain as GST fusion proteins (from now on termed as Arm wt and Arm E193K). GST-only served as control, to detect false positives caused by unspecific binding to the affinity tag or matrix. By western-blotting, we noticed that Arm E193K variant binds total DRP1 as well as the active form, phospho-616 DRP1 (Taguchi et al., 2007) with increased efficiency (Figures 7C,D). To further test the impact of E193K variant on the LRRK2 binding feature, we isolated full-length FLAG-LRRK2 WT and E193K protein from transfected N2A cells and studied LRRK2 binding to DRP1 and mitofusin-2, a protein involved in mitochondrial fission (Ishihara et al., 2004) previously proposed as LRRK2 interactor (Stafa et al., 2014). Again, E193K variant demonstrated a robustly increased affinity for DRP1 while the binding with mitofusin-2 was not affected (Figures 7E,F and Supplementary Figures S1C–E). Next, we studied the LRRK2-DRP1 complex in the presence of MPP+ (1 mM). Interestingly, we found that upon MPP+ the binding between LRRK2 E193K and DRP1 was significantly impaired while the LRRK2 WT/DRP1 complex remained stable (Figures 7E,F). Given the key role played by DRP1 in mitochondrial fission and the impact of E193K variant on LRRK2-DRP1 complex, we investigated the mitochondrial response to MPP+ in healthy and E193K fibroblasts upon over-expression of GFP or GFP-DRP1. Living fibroblasts were treated with MPP+ (1 mM, 24 h) or vehicle and then incubated with MitotrackerRed (Figure 8A and Supplementary Figure S3). MPP+ insult caused mitochondria fragmentation in GFP or GFP-DRP1 expressing WT fibroblasts, as reflected by reduced interconnectivity and length of mitochondria. By contrast, in GFP-expressing E193K cells MPP+ did not induce overt mitochondrial fission. We observed a similar outcome in HeLa cells upon over-expression of LRRK2 variants and MPP+ treatment (Supplementary Figure S4). Interestingly, we noticed that GFP-DRP1 over-expression correlated with mitochondrial fission upon MPP+ insult in E193K fibroblasts (Figures 8B,C and Supplementary Figure S3). Altogether, these data suggest that the E193K variant affects mitochondrial fission upon MPP+ exposure by influencing LRRK2-DRP1 complex.

## Discussion

**Figure 9:**
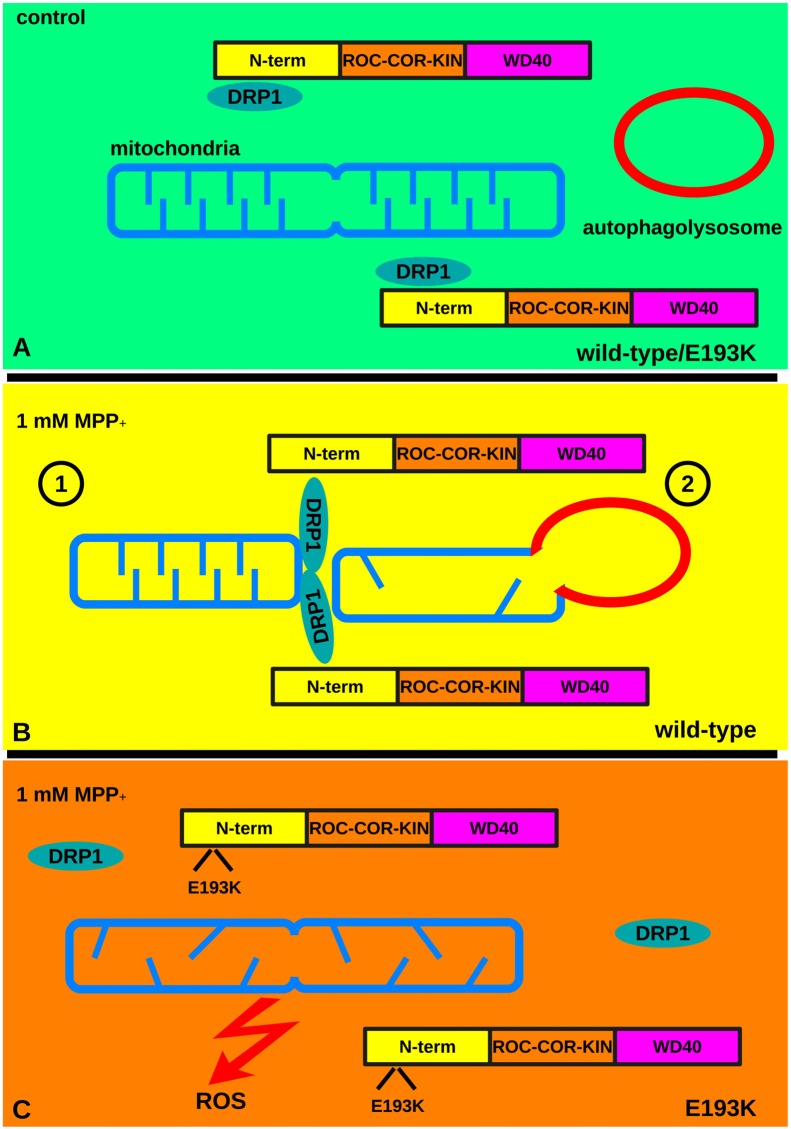
The E193K variant prevents mitochondrial fission upon MPP+ treatment. **(A)** Under basal conditions mitochondria undergo continuous cycles of fusion and fission. Fission requires the activity of DRP1. LRRK2 binds to DRP1 and increases DRP1 recruitment to mitochondria. **(B)** Under noxious stimuli, WT cells react via the LRRK2-DRP1 complex that allows mitochondrial fission (1). Eventually, damaged mitochondria are eliminated via autophagy (2) limiting the production of ROS. **(C)** In the presence of a toxic insult the LRRK2 E193K-DRP1 complex is partially destabilized. This leads to an incomplete fragmentation of mitochondria that prevents the clearance of damaged organelles and increases the production of ROS.

We described here the functional impact of the E193K substitution on LRRK2 identified in one Italian family with three PD-affected siblings. Despite the fact that the mutation is very rare and likely a private mutation, we believe that an extensive functional characterization of this variant could help in elucidating LRRK2 tasks. This protein has a key role in PD pathogenesis, influences autophagy and mitochondrial function and may be an important therapeutical target for idiopathic PD (Wang B. et al., 2016). In particular, our data bring further support to the hypothesis that LRRK2 is implicated in mitochondrial homeostasis. Accumulating literature has demonstrated the link among mitochondrial dysfunction, oxidative stress and the pathogenesis of multiple neurodegenerative disorders including PD (Bose and Beal, 2016). This does not come unexpectedly: mitochondrial function is essential to cellular processes such as energy production and ions regulation. Furthermore, neurons do have special energetic needs to fuel axonal and dendritic transport, the release and re-uptake of neurotransmitters and vesicle trafficking at synapses. Mitochondria go through continuous cycles of fusion and fission. Various PD-associated proteins are involved in the regulation of mitochondrial dynamics. PINK1/Parkin regulates the ubiquitination and degradation of mitofusin 2 and DRP1 while LRRK2 increases DRP1 recruitment to mitochondria (Luo et al., 2015). LRRK2 PD mutations increase vulnerability to mitochondrial stress (Saha et al., 2009; Wang et al., 2012) but the precise mechanism underlined has not been fully understood yet. We describe here that the E193K variant has no major impact on mitochondrial basal activity but instead increases susceptibility to MPP+. This comes in good agreement with the GxE theory underlying PD. The exposure to stressors and genetic factor(s) together can trigger the degenerative cascade (Bose and Beal, 2016). The E193K substitution falls within the armadillo repeats domain. Armadillo repeats fold together into a superhelix, which constitutes a versatile platform for multiple protein interactions (Tewari et al., 2010). The substitution of a negatively charged residue, E, with a positively charged one, K, is strongly predicted to influence the biochemical properties of the N-terminal Armadillo domain of LRRK2. We found that the E193K substitution alters the binding between LRRK2 and DRP1 (Wang et al., 2012). Among its several functions, it is well established that DRP1 executes mitochondrial fission (Chang and Blackstone, 2010). In particular, it is a crucial mediator of mitochondrial fission upon MPP+ treatment (Wang et al., 2011). It has been suggested that upon initial fission, DRP1 complex must be removed from the mitochondrial outer-membrane to allow the full separation of the formed segment, i.e., fragmentation. Otherwise, DRP1 exerts an inhibitory action due to the occupancy of fission sites, sequestration of other effectors or steric hindrance (Zhao et al., 2011; Wang W. et al., 2016). Accumulating evidence suggests that mitochondrial fragmentation precedes mitophagy. In particular, the activity of DRP1 correlates well with mitochondrial fission and autophagy: increased DRP1 activity facilitates mitochondrial elimination under noxious stimuli and the expression of a DRP1 dominant negative isoform impaired mitophagy (Arnoult et al., 2005). These observations suggest that fission is instrumental for mitochondrial fragmentation, which might be seen as a rescue mechanism that permits the segregation and elimination of dysfunctional mitochondria. LRRK2 directly interacts with DRP1 and increases DRP1 recruitment to mitochondria (Wang et al., 2012). Given the reduced affinity for DRP1 demonstrated by E193K LRRK2 upon MPP+ treatment, it can be argued that E193K substitution may partially destabilize the DRP1 complex at mitochondria in the presence of a toxic insult. This may lead to incomplete fragmentation of mitochondria that prevents the autophagic clearance of damaged organelles (Gomes et al., 2011). Accordingly, while in control fibroblasts mitochondria underwent evident fragmentation, we consistently observed the appearance of segmented, highly branched mitochondria in E193K cells upon MPP+. Damaged mitochondria are a source of oxidative stress (Twig et al., 2008): not surprisingly we noticed increased ROS production and cellular toxicity in E193K cells upon MPP+. Interestingly, G2019S fibroblasts were characterized by increased susceptibility to MPP+ as compared to E193K line in terms of viability and mitochondrial respiration but demonstrated mitochondrial fragmentation as WT lines did. Thus, G2019S and E193K might affect mitochondrial response to toxins acting on different pathways. Recently, it has been reported G2019S mutation may alter mitochondrial calcium currents (Verma et al., 2017). Our data indicate that E193K variant alters the dynamics of LRRK2-DRP1 complex upon MPP+ exposure. Thus we hypothesize that the E193K variant interferes with the stress-induced turnover of the LRRK2-DRP1 complex thus impairing autophagic removal of damaged mitochondria and eventually increasing cellular sensitivity to noxious stimuli (Figure 9). We have previously demonstrated that LRRK2 acts as a scaffold at the presynaptic site where it influences vesicle recycling via protein-protein interactions (Piccoli et al., 2011, 2014; Bedford et al., 2016; Carrion et al., 2017). Furthermore, LRRK2 binds and phosphorylates Rab proteins, the master regulators of membrane fusion (Steger et al., 2016). Thus, one intriguing hypothesis is that LRRK2 acts as a molecular hub that organizes structural and regulatory elements to control proper trafficking of different membranous structures, from synaptic vesicles to mitochondria. We are well aware of the potential bias due to the intrinsic features of a given primary fibroblast line. However, we succeeded in recapitulating our key findings obtained in primary fibroblasts in heterologous lines upon over-expression of the LRRK2 E193K variant. Thus, we are confident that our study addressed specifically the biological effect of the E193K substitution. The E193K variant might affect LRRK2 function via perturbation of its physiological network of interactors. It has been shown that several LRRK2 variants as A1442P, R1441C and the G2385R affect LRRK2 steady-state levels (Greene et al., 2014; Rudenko et al., 2017): thus it deserves further investigation the possibility that E193K variant might impact LRRK2 protein turn-over. Intriguingly, at the other side of the protein, the G2385R point mutation within the WD40 domain perturbs LRRK2 biochemical features and influences LRRK2 functions (Jorgensen et al., 2009; Rudenko et al., 2012; Piccoli et al., 2014; Carrion et al., 2017). Thus, the regulation of the LRRK2 protein complex is emerging not only as a key factor in its physiological and pathological function but could arise as a target for future therapeutic approaches besides the LRRK2 kinase activity.

## Author Contributions

MPC, MG, LS, IR, FP, AB, LC and MWH performed experiments. MPC, LP, NT, CT, JEL and CJG analyzed data. CM and GPezzoli visited patients. GPiccoli, IP, EG, MWH, SD and SG designed experiments and wrote the article.

## Conflict of Interest Statement

The authors declare that the research was conducted in the absence of any commercial or financial relationships that could be construed as a potential conflict of interest.

## Footnotes

^1^http://www.pymol.org/
