# Supplementary material for: The LRRK2 Variant E193K Prevents Mitochondrial Fission Upon MPP+ Treatment by Altering LRRK2 Binding to DRP1
Source: Front Mol Neurosci. 2018 Feb 28;11:64. doi: 10.3389/fnmol.2018.00064 (PMC5835904; doi:10.3389/fnmol.2018.00064)
Supplement: Supplementary file 1 [file Data_Sheet_1.doc]

**SUPPLEMENTARY MATERIAL**

**Supplementary text**

**Cases Description**

G-0502 (1853)

In this patient, a 64-year-old man, PD presented at 47-year old (2000) with resting tremor, bradykinesia and rigidity on the left arm. Diagnosis of PD was done after one year and Dopamine-Agonist and MAO-B inhibitor treatment was started with good response. Brain RMN was unremarkable. Progression was slow. He started levodopa afer 7 years of disease duration with good response. Dyskinesias and on-off fluctuation started at 10 years of disease-duration (2010): UPDRS motor score = 11; Hoehn and Yahr stage = 1. In 2012 (12 years from onset), he developed a severe osteomyelitis causing tetraparesis which was slowly and partially improving, worsening the movement disorder. Cognitive functions had been always preserved. He underwent extensive neuropsychological assessment at 15 years of disease duration (2015) and showed preserved cognitive functions as well as other behavioural conditions. Depression, apathy and sleep problems were present. At last examination (2016), UPDRS motor score was 36 and H/Y stage 4.

G-1350 (3859)

This patient was a 56-year-old man. PD presented at 48-year old (2009) with resting tremor, bradykinesia and rigidity on the right arm. Diagnosis of PD was done after one year and Dopamine-Agonist treatment was started with good response. Brain RMN was unremarkable, while dopamine transporter SPECT imaging confirmed the clinical diagnosis of PD (2011). During time, progression was slow. He started levodopa after 3 years of disease duration with good response (2012). He underwent extensive neuropsychological assessment (2013) which did not show any problems. At last examination (2014), UPDRS motor score was 11 and H/Y stage 2, without dyskinesias or motor fluctuations.

G-1677 (6070)

This patient lives in Canada and is followed by the Movement Disorders Centre, Toronto Western Hospital, University of Toronto, Canada (Dr. A Fasano). He developed PD at 47 years of age (1994) with left leg stiffness and bilateral resting tremor. Progression was slow and response to levodopa was good. In the past he had hypomania, hypersexuality and gambling with DopaAgonist. He underwent extensive formal NPS assessment in 2006 that resulted substantially in the normal range.

Because of severe motor fluctuations and dyskinesias, he underwent right STN DBS in 2008. Due to psychiatric concerns he only underwent an unilateral procedure and then he got left pallidotomy in March 2012. The IPG was replaced in August 2012 (Activa). He also presents axonal neuropathy and right carpal tunnel syndrome diagnosed in 2010. At last examination in Dec 2016, UPDRS motor score was 22 (med on/stim on) and H/Y stage was 2.5 without dyskinesias or motor fluctuations.

**Supplementary figures captions**

**Supplementary figure 1. Biochemical investigation of E193K variant.** (A) HEK293 cells expressing Strep-FLAG-LRRK2 wild-type, Strep-FLAG-LRRK2 E193K and Strep-FLAG-LRRK2 G2019S variant were solubilized and processed for western-blotting to appreciate LRRK2 phosphorylation level at Ser935. (B) The graph reports P-Ser935 level, expressed as optical density and normalized versus total LRRK2 amount. Data are shown as mean ±S.E, n=4. *** p<0.001 versus wild-type. (C) HEK293 cells expressing Strep-FLAG-LRRK2 wild-type, Strep-FLAG-LRRK2 E193K and Strep-FLAG-LRRK2 G2019S variant were solubilized and processed for Streptavidin immunopurification. We evaluated the extent of DRP1and mitofusin 2 binding to LRRK2 by measuring the amount of DRP1 and mitofusin 2 protein co-precipitating with Strep-FLAG LRRK2 variant. (D) The graph reports the amount of DRP1 recovered in Strep-Flag-LRRK2 immunoprecipitates. Data were normalized to the amount of LRRK2 variant immunoprecipitated and expressed as mean ± S.E. (n=4); * p<0.05 versus wild-type .(E) The graph reports the amount of mitofusin-2 recovered in Strep-FLAG immunoprecipitates. Data were normalized to the amount of LRRK2 variant immunoprecipitated and expressed as mean ± S.E. (n=4). (F) Oxygen consumption assessed by high resolution respirometry of primary fibroblasts after 24 hours treatment with 1 mM MPP+. The graph reports routine activity (routine respiration), complex I and complex II – dependent activity (upon treatment with malate, glutamate and succinate) and maximal activity (maximal uncoupled respiration after FCCP injection) upon MPP+ normalized to control condition. Data show means ± S.E., n= 4; **, ***p<0.01, 0.001 versus control conditions, same genotype.

**Supplementary figure 2. Mitochondrial morphology is impaired in E193K fibroblasts.** Transmission electron microscopy from cryo-fixed, freeze-substituted fibroblast cultures. Untreated wild type, G2019S or E193K fibroblasts showed elongated, partly branched mitochondria with correctly arranged cristae. Upon treatment with MPP+, wild type and G2019S fibroblasts appeared consistently fragmented (and locally highly swollen), showing reduced and disorganized cristae. Arrowheads point to fragmented mitochondria. By contrast, E193K fibroblasts were regularly characterized by conspicuously segmented, highly anastomosing mitochondrial networks and altered cristae morphology. Asterisks point to segmented mitochondria. Segmented mitochondria appeared only in E193K cells upon MPP+ and occurred in more than 70% of the cells analysed. Representative images are shown from 3 independent cell culture experiments (20 cells genotype/treatment/experiment). Scale bar=2µm.

**Supplementary figure 3. Analysis of mitochondrial morphology.**  Primary fibroblasts obtained from healthy individuals and an E193K carrier were transfected with GFP or GFP-DRP1, treated with 1 mM MPP+ for 24 hours and then incubated with MitotrackerRed to investigate mitochondrial morphology. Scale bars= 10 μm.

**Supplementary figure 4. Analysis of mitochondrial morphology.** HeLa cells over-expressing wild-type or E193K GFP-LRRK2 variants were treated with 1 mM MPP+ for 24 hours and then incubated with MitotrackerRed to investigate mitochondrial morphology. Scale bar= 10 μm. In control condition mitochondria were organized in diffuse filaments. Upon MPP+, mitochondrial filaments disappeared. Instead we detected the appearance of small mitochondrial clusters (arrows). E193K expressing cells were characterized by an impaired number of mitochondrial clusters and by residual mitochondrial filaments (arrowhead). The number of mitochondrial clusters appearing in cells are indicated. Data are expressed as mean ± S.E. (n=3, 5-6 cells/experiment). ** p<0.01 versus wild-type.
