# Supplementary figures and images for: The LRRK2 Variant E193K Prevents Mitochondrial Fission Upon MPP+ Treatment by Altering LRRK2 Binding to DRP1
Source: Front Mol Neurosci. 2018 Feb 28;11:64. doi: 10.3389/fnmol.2018.00064 (PMC5835904; doi:10.3389/fnmol.2018.00064)

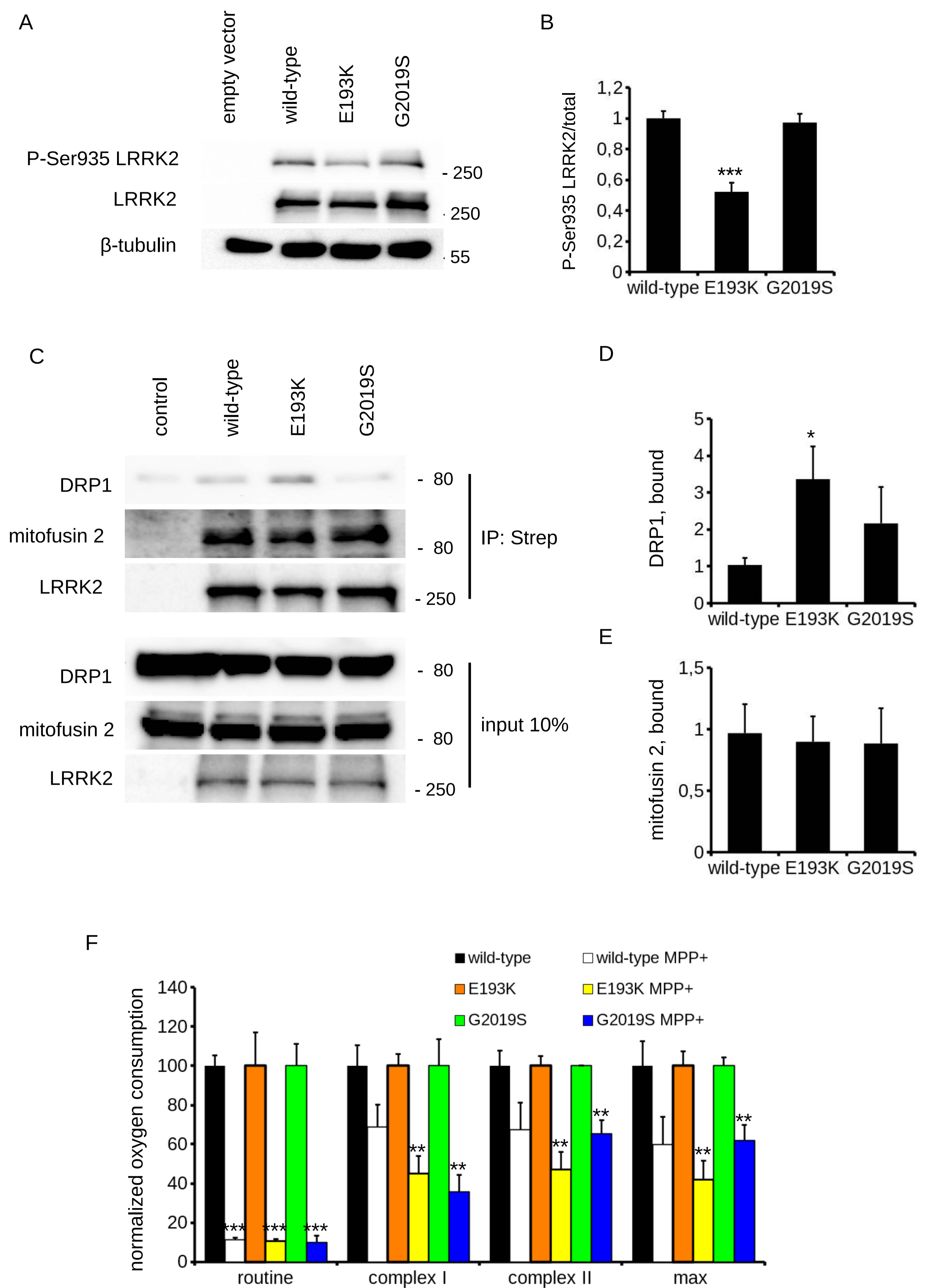

Supplementary figure 1

control

1 mM MPP+

wild-type

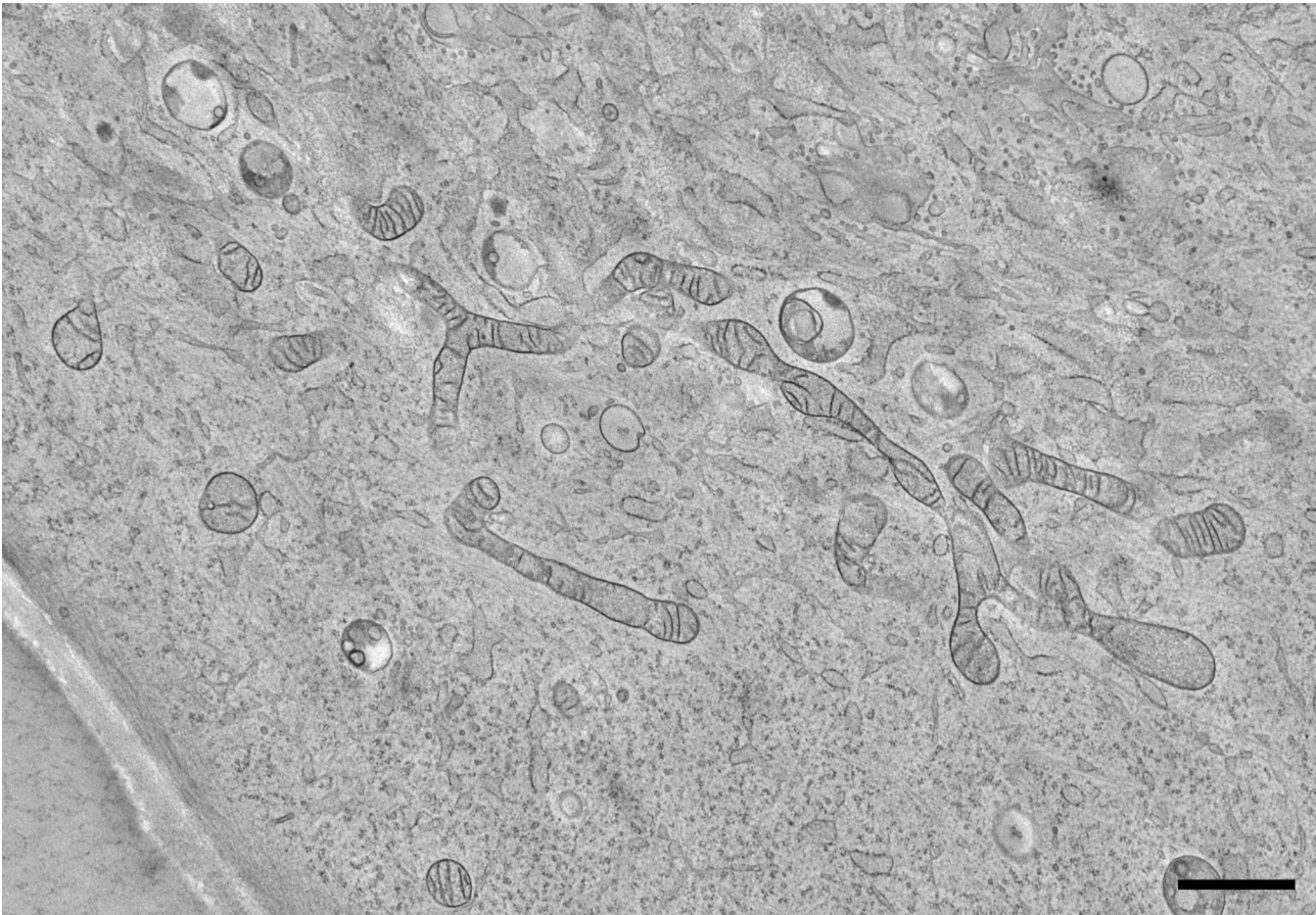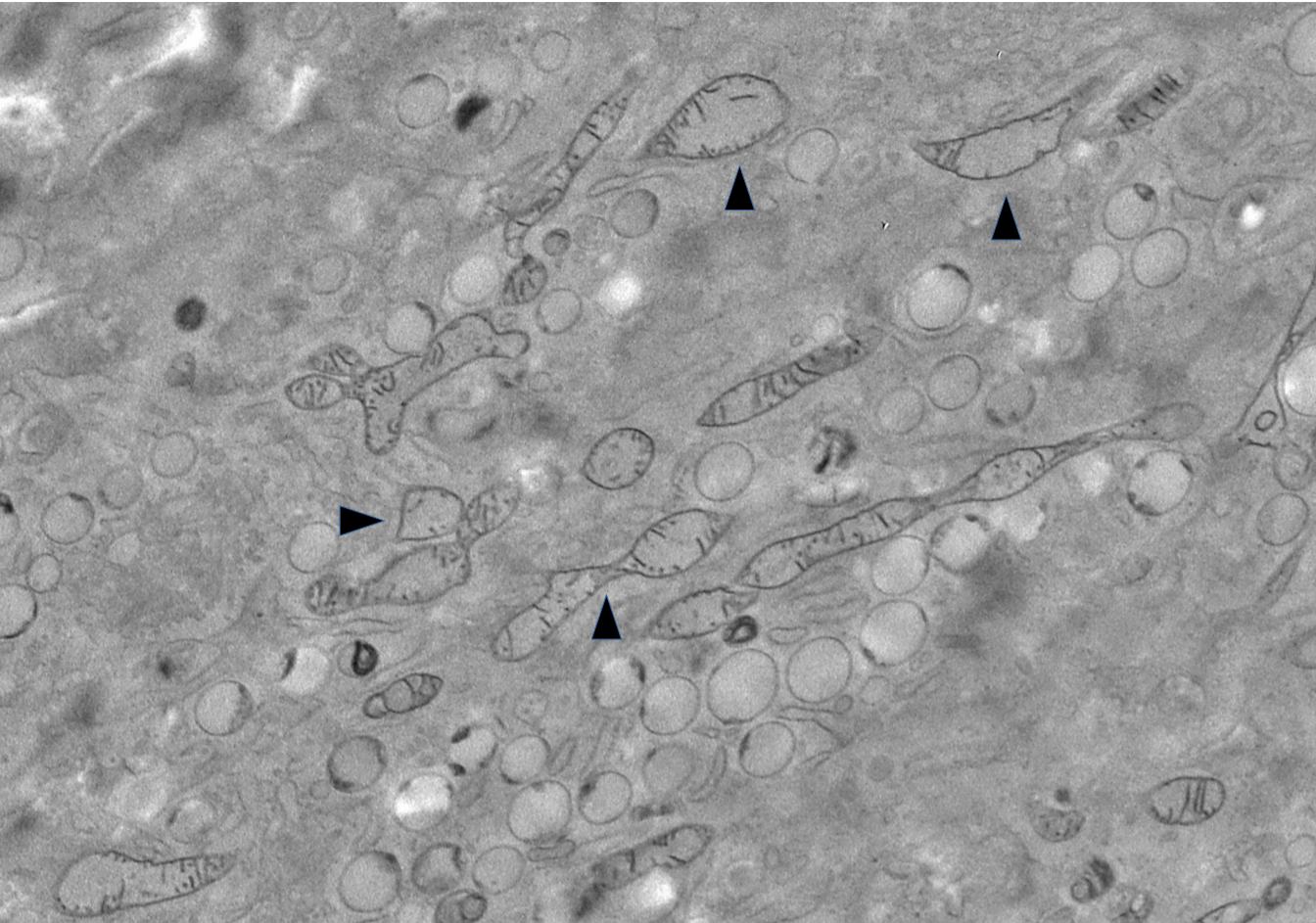

E193K

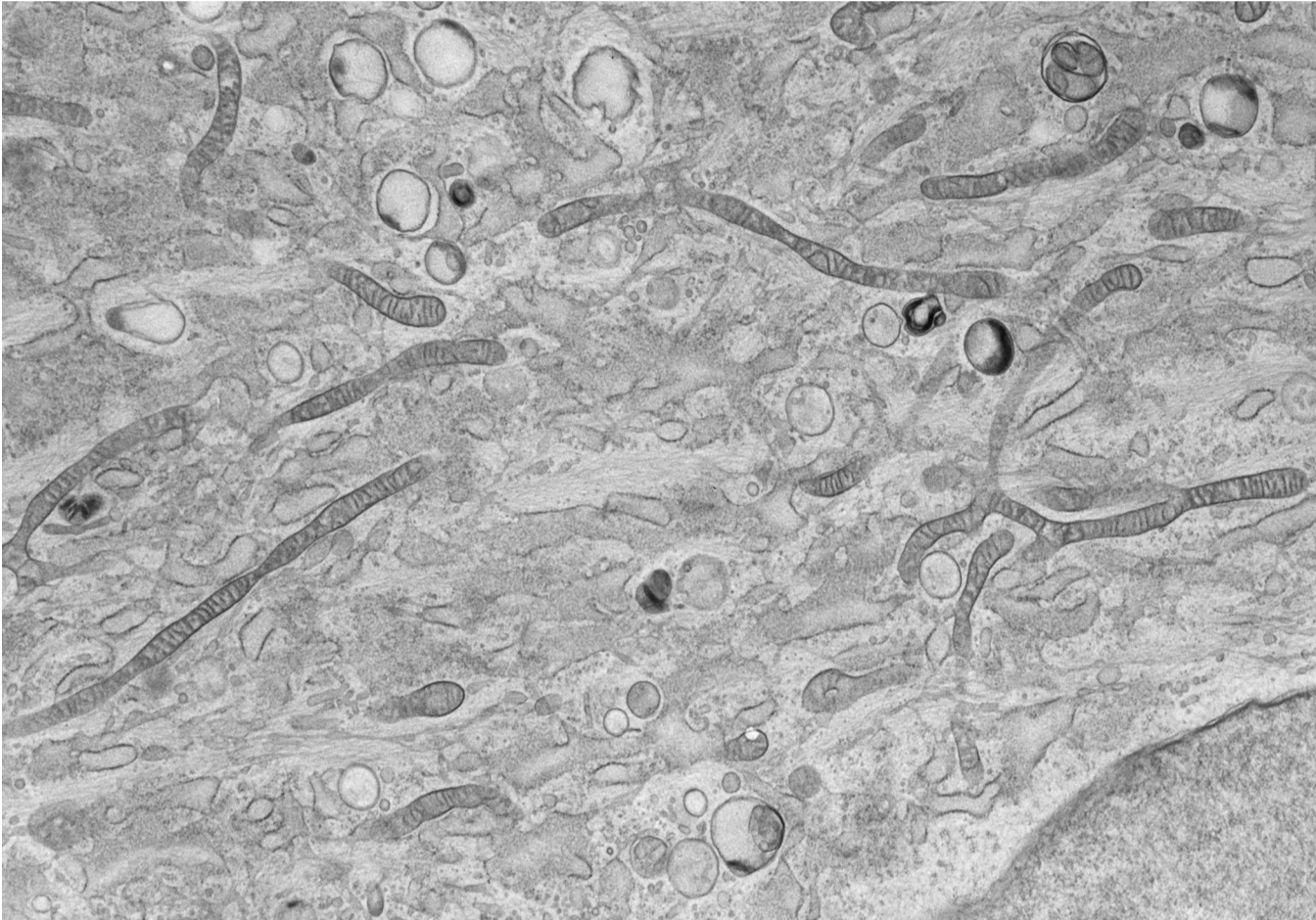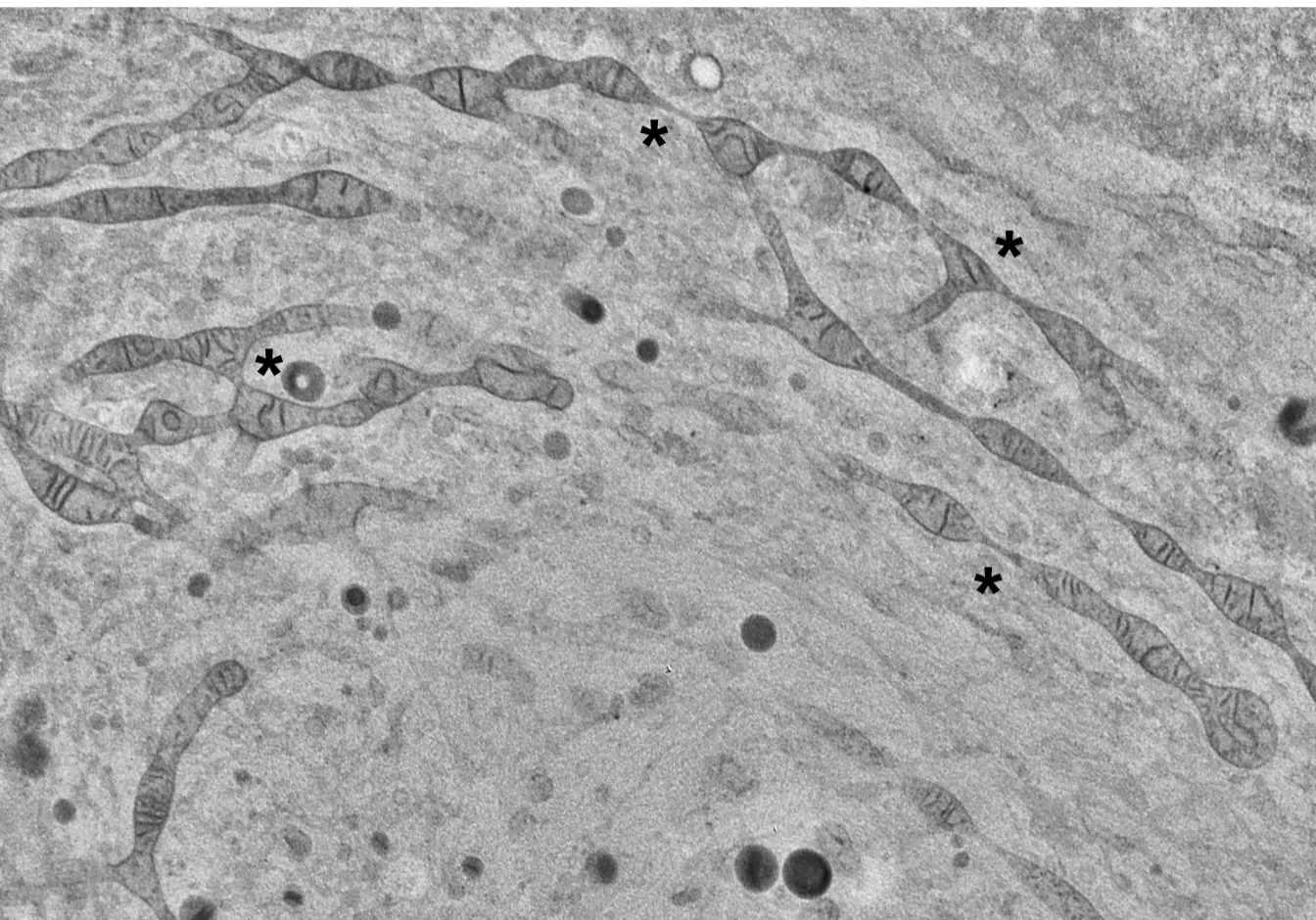

G2019S

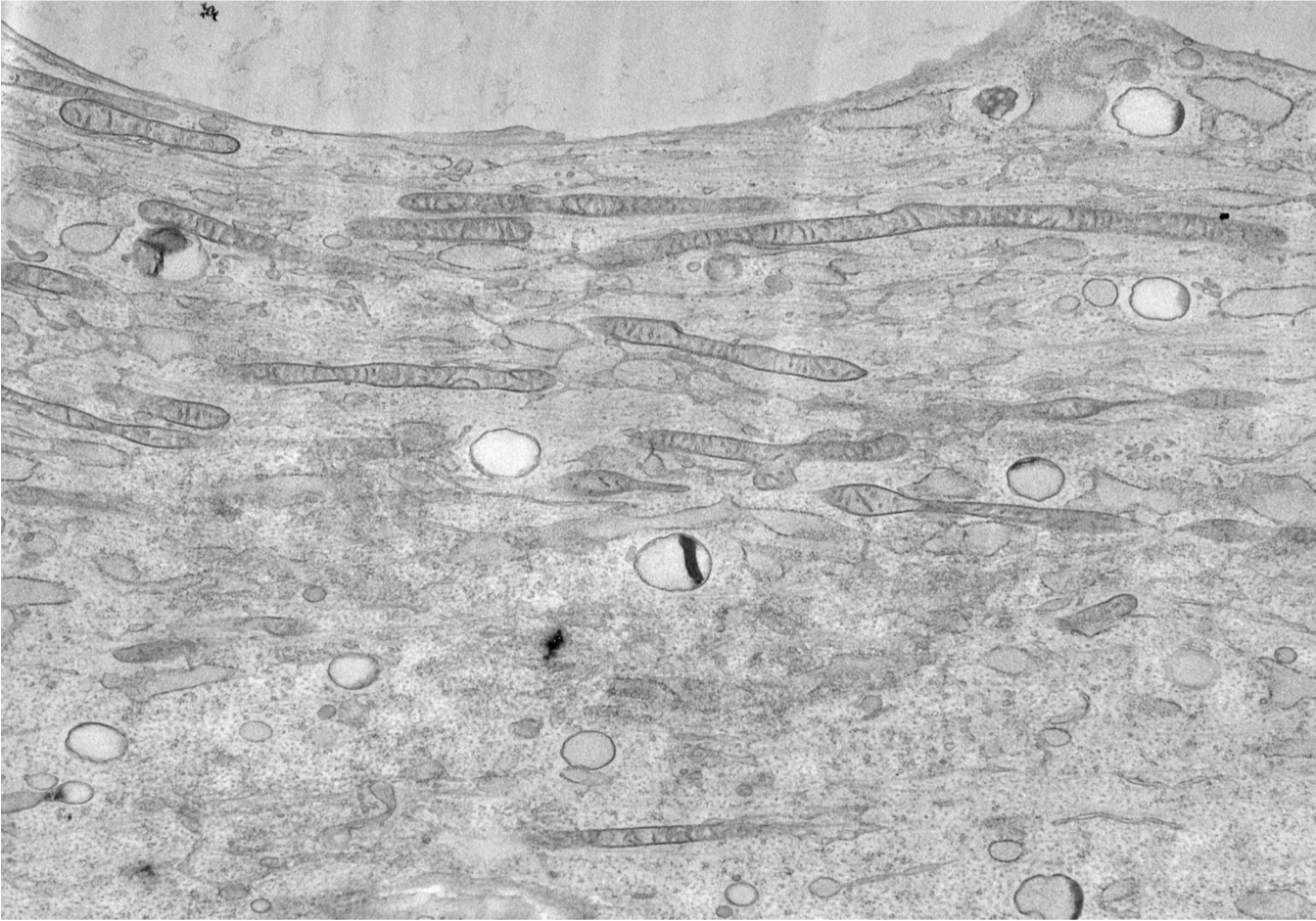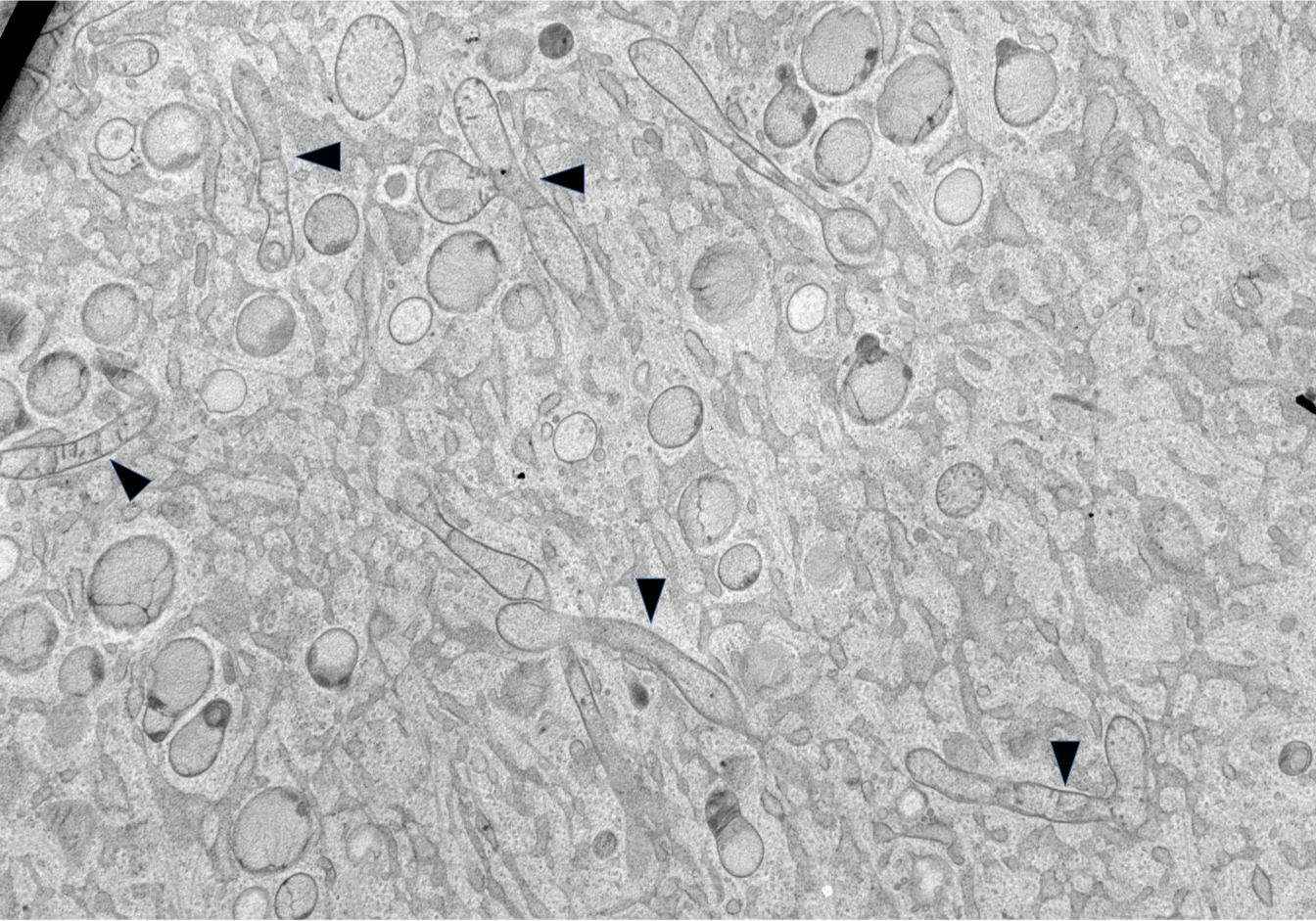

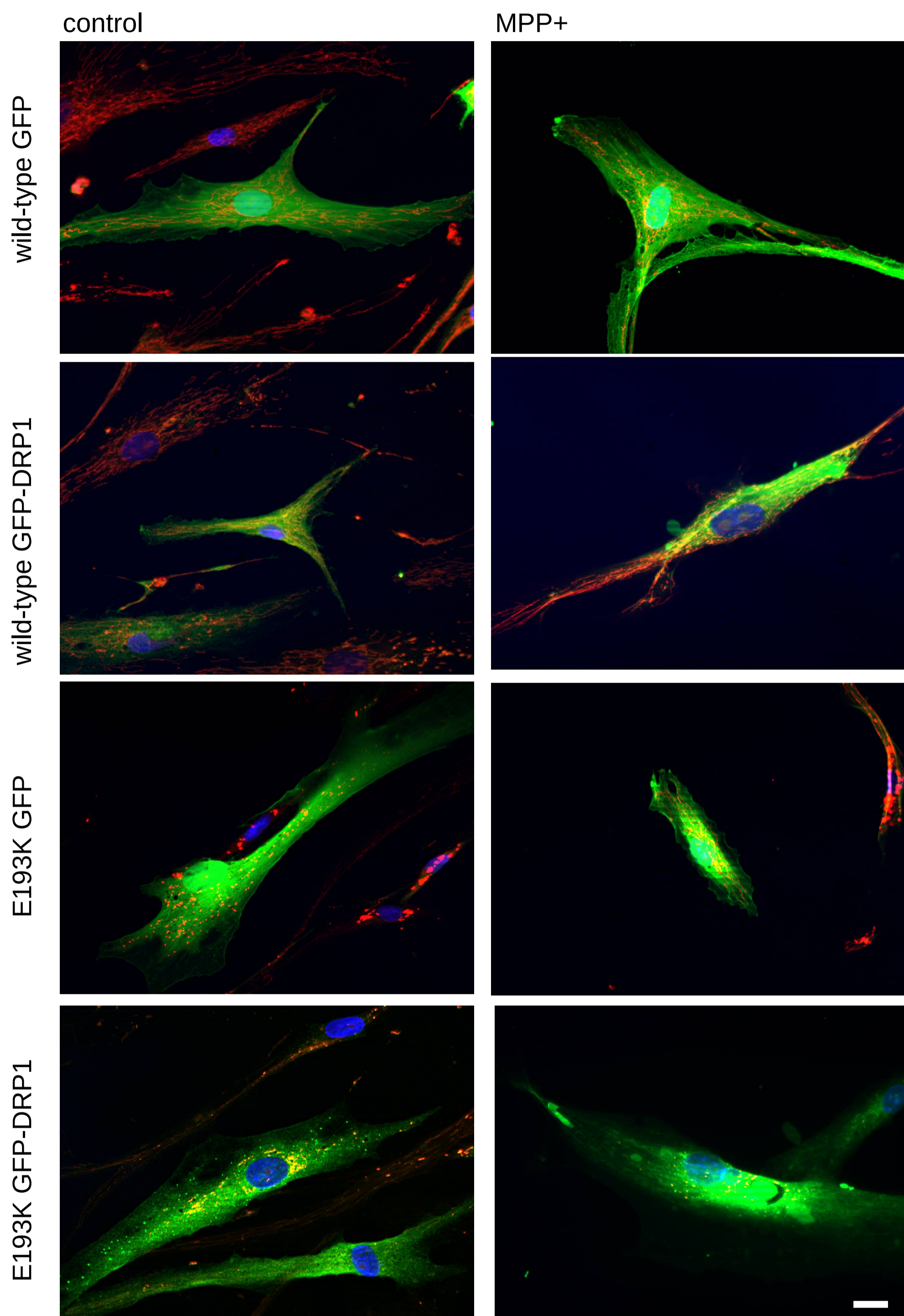

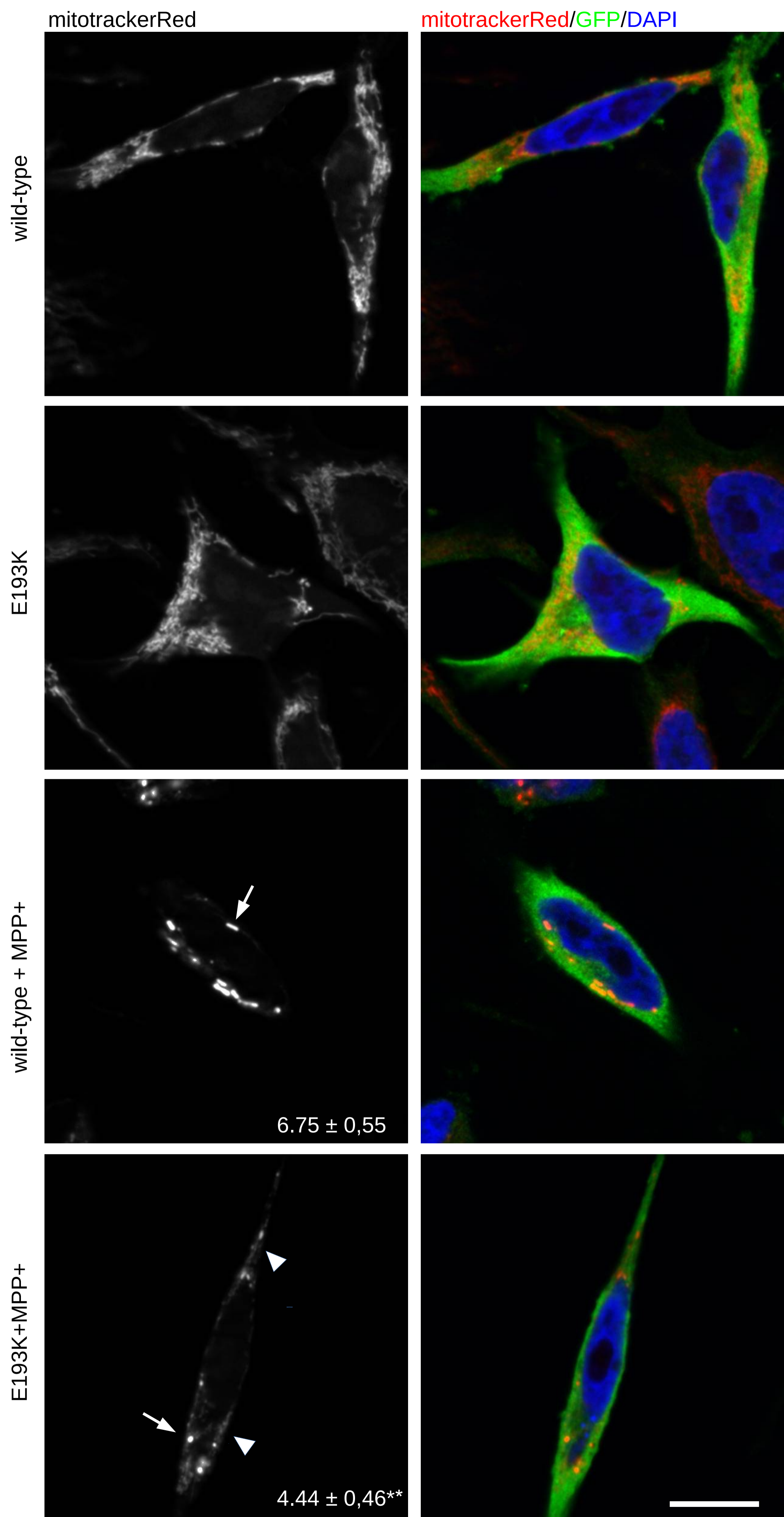

Supplementary figure 4

Supplement: Supplementary file 2 [file Image_1.pdf]
